# The Gut Microbiota–Muscle Axis: *Lactobacillus* as a Potential Modulator of Skeletal Muscle Health

**DOI:** 10.1155/bmri/7133290

**Published:** 2026-09-22

**Authors:** Bencia Janet, Rutuparna Jena, B. S. Satish Rao, Budheswar Dehury

**Affiliations:** ^1^ Department of Radiation Biology and Toxicology, Manipal School of Life Sciences, Manipal Academy of Higher Education, Manipal, India, manipal.edu; ^2^ Department of Bioinformatics, Manipal School of Life Sciences, Manipal Academy of Higher Education, Manipal, India, manipal.edu

**Keywords:** diabetes mellitus, exercise, gut microbiota, *Lactobacillus*, obesity, sarcopenia, skeletal muscle

## Abstract

Skeletal muscle constitutes one of the largest tissue compartments in the human body and plays fundamental roles in locomotion, posture maintenance, and metabolic regulation. Muscle homeostasis requires a precise balance between protein synthesis and degradation pathways; disruption of this equilibrium leads to pathological conditions, including sarcopenia and muscle atrophy, which frequently coexist with metabolic disorders such as obesity, Type 2 diabetes mellitus, and metabolic syndrome. Recent advances have identified the gut microbiota as an important regulator of skeletal muscle health, positioning probiotics as potential therapeutic interventions. Among probiotic candidates, *Lactobacillus* species have been associated with improvements in muscle health through pleiotropic mechanisms, including attenuation of inflammatory signaling, enhancement of mitochondrial biogenesis, modulation of muscle protein turnover, and restoration of the intestinal microbial balance by enriching beneficial bacteria while suppressing pathogenic species. These multifaceted effects translate to improved exercise performance, amelioration of metabolic dysfunction, and prevention of muscle wasting. This review comprehensively evaluates the current evidence supporting *Lactobacillus* supplementation as a strategy to increase skeletal muscle health and function, examines the underlying molecular mechanisms, and identifies critical research priorities, including strain‐specific characterization, well‐controlled human clinical trials, and mechanistic investigations, to optimize probiotic‐based interventions for muscle‐related disorders.

## 1. Introduction

Skeletal muscle, the body′s most abundant and crucial tissue comprising 40%–50% of body weight, maintains posture, balance, and movement while it metabolizes energy substrates and regulates body temperature [1, 2]. Preserving skeletal muscle mass and strength is essential for maintaining the body′s energy balance and the ability to perform daily tasks [3]. The growth and maintenance of skeletal muscle depend on the equilibrium between protein synthesis and protein breakdown [4]. An imbalance between protein synthesis and breakdown can contribute to disorders such as muscular atrophy [1].

Sarcopenia or skeletal muscle atrophy is characterized by progressive muscle loss, which leads to reduced muscle function and strength over time [5, 6]. It is often associated with various metabolic disorders, such as diabetes, obesity, nonalcoholic fatty liver disease (NAFLD), and cancer [7–11]. The insulin‐like growth factor 1 (IGF1)–protein kinase B (Akt) pathway plays a pivotal role in regulating muscle growth. Upon binding to its receptor, IGF1 activates phosphatidylinositol‐3‐kinase (PI3K) and Akt [12], promoting muscle protein synthesis [13, 14]. Conversely, muscle atrophy is mediated by the ubiquitin–proteasome system (UPS), where Atrogin‐1 and Muscle RING‐finger protein 1 (MuRF1) facilitate protein degradation. These proteins are considered potential therapeutic targets for combating muscle loss. Additionally, ghrelin, a hormone, has been shown to protect against muscle atrophy by increasing Akt phosphorylation [12]. Maintaining muscle health requires a combination of resistance exercise and a protein‐rich diet. Regular physical activity strengthens the musculoskeletal system, improves strength and endurance, and mitigates age‐related muscle loss (sarcopenia) [4, 15]. However, excessive or prolonged exercise can induce structural damage and acute inflammation, a condition termed exercise‐induced muscle damage (EIMD) [16].

In recent years, the role of the gut microbial community in various diseases has been studied, and the “gut muscle axis” defines the correlation between the gut microbiota and muscle health [17]. The human gut microbiota consists of 10–100 trillion metabolically active microorganisms that play crucial roles in the physiology of the host [18, 19]. A balanced gut microbiota state supports the health of the host through the production of microbial metabolites [20]. Conversely, dysbiosis involves a reduction in microbial diversity and an alteration in the abundance of specific microbial taxa and their metabolites, with an increase in harmful microbes in the gut [21, 22]. Dietary shifts, for example, increased consumption of ultraprocessed foods and reduced dietary fiber intake, have triggered alterations in the composition of the human gut microbiota and modified the intestinal microenvironment [23]. To maintain the homeostasis of the gut microbiota, probiotics are introduced [24]. Probiotics, as defined by the International Scientific Association for Probiotics and Prebiotics (ISAPP), are live microorganisms that, when administered in adequate amounts, confer a health benefit to the host. These viable microbes include bacteria and some fungi that are naturally present in the human intestine and are found in fermented foods. These compounds, when administered in appropriate amounts, provide beneficial effects to the host [25–27]. It regulates several diseases by modulating the gut microbial composition and producing metabolites, such as branched‐chain amino acids (BCAAs) and short‐chain fatty acids (SCFAs) [14, 23]. The probiotic strains used to enhance skeletal muscle health include *Lactobacillus*. Lactic acid bacteria (LAB), particularly *Lactobacillus* species, are gram‐positive, anaerobic, and nonspore‐forming bacteria that are naturally found in the human gut [28]. They are widely used as probiotics, either alone or in combination, owing to their beneficial health effects. These bacteria exhibit anti‐inflammatory, anticancer, and antioxidant properties. Additionally, they support immune function, maintain the gut microbial balance, protect against gastrointestinal infections, and contribute to cholesterol‐lowering effects [29].

Gut microbial dysbiosis facilitates the passage of endotoxins and other microbial products in the circulation, which in turn triggers innate immunity. This leads to low‐grade systemic inflammation and related metabolic and muscular diseases [22]. High concentrations of immune cells are present in the intestine, and these cells are influenced by the gut microbiota. SCFAs produced in the gut with the help of solute transporters are transported into the blood circulation [30]. These compounds are absorbed into cells and can suppress the activity of Class I and II HDACs and Type III HDACs, thereby increasing protein acetylation. SCFA‐induced acetylation plays a crucial role in the generation of IL‐10–producing B cells and hence promotes IL‐10+ regulatory T‐cell activity. In addition, it serves as a nutrient for B cells and enhances B‐cell activation during active immune responses. The conversion of SCFAs to acetyl‐CoA enables the formation of long‐chain fatty acids, which in turn leads to plasma B‐cell differentiation. It is also involved in increased IgG and IgA production. To regulate the gut microbiota and prevent dysbiosis, SCFAs increase the number of IgA‐coated bacteria in the gut [30]. Muscle cells release soluble myokines, such as IL‐6, IL‐7, IL‐15, and LIF, which modulate the immune system [30, 31]. The downstream communication of IL‐15 occurs via the Janus kinases 1, JAK 3, STAT3, and STAT5 pathways plays a role in the development and maintenance of immune cells, including the proliferation, activation of natural killer (NK) cells, the survival and modulation of T cells, and the proliferation of B cells. In skeletal muscles, AMPK acts as a sensor of intracellular energy levels and is pivotal for exercise‐induced IL‐15 production. A shift in the levels of IL‐15 has been observed in patients with sarcopenia. Chronic low‐grade inflammation in sarcopenia is associated with the activity of IL‐6, which has both proinflammatory and anti‐inflammatory properties. Similarly, the levels of IL‐7 and its downstream signaling are mediated by Jak1 and ‐3 activation, which results in the phosphorylation of STAT‐molecules, shown to be decreasing with age and is a link between immune activity and aging. Sarcopenic conditions involve the upregulation of the JAKSTAT, ERBB, and IL‐2/4 signaling pathways along with macrophage‐rich inflammation in skeletal muscles [32, 33].

Skeletal muscle atrophy and exercise‐induced muscle loss are linked to gut microbial dysbiosis (the modulation of the gut microbial community). Bacteria such as *Lactobacillus*, *Bifidobacterium*, *Proteobacteria*, *Akkermansia*, *Clostridium*, *Ruminococcacae*, *Roseburia*, *Bacteroides*, *Prevotella*, *Faecalibacterium*, and *Firmicutes* are associated with skeletal muscle health. Additionally, SCFAs such as acetate, propionate, and butyrate, with butyrate being the major SCFA, improve skeletal muscle health [34–36]. Research has indicated that moderate exercise, structured training, nutritional supplementation, or probiotics can mitigate muscle loss and increase exercise capacity [14]. These interventions positively affect the musculoskeletal system by improving muscular strength, endurance, and flexibility while reducing the risk of sarcopenia [15]. Additionally, regular resistance training has been shown to enhance exercise performance and prevent sarcopenia [4]. In light of these findings, this review provides a comprehensive examination of *Lactobacillus* supplementation and its effects on exercise performance, muscle‐related metabolic disorders, and muscle atrophy.

## 2. Method

### 2.1. Search Strategy

The literature search was performed using the platforms PubMed and Google Scholar. Articles relevant to the title of this review are identified. The keywords used included (1) *Lactobacillus* and muscle atrophy or sarcopenia, (2) *Lactobacillus* and diabetes and obesity, (3) *Lactobacillus* and exercise, and (4) *Lactobacillus* and NAFLD or metabolic syndrome. The literature search is limited to publications from January 2015 to June 2026.

### 2.2. Inclusion Criteria

All the research articles based on animal studies, in vitro experiments, and clinical trials, along with recent review papers, were chosen for further review. Only English articles were further screened for compatibility.

### 2.3. Exclusion Criteria

**Figure 1:**
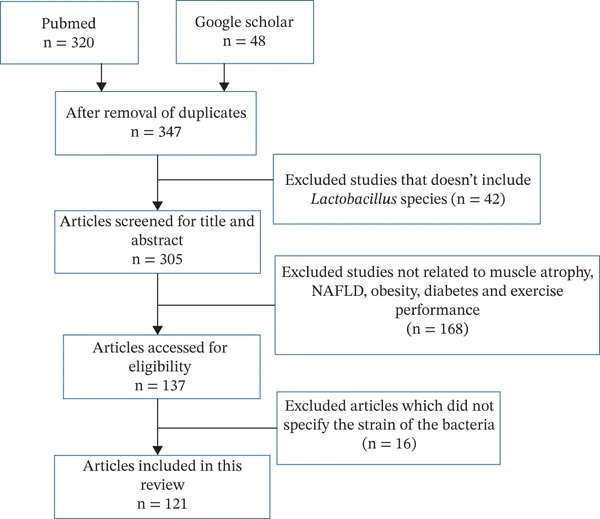
The proposed workflow was adopted for screening the articles to write the present review.

Studies that did not mention the strain of the probiotic bacteria were excluded, and metabolic disorders other than NAFLD, obesity, and diabetes were excluded. The results of the step‐by‐step screening of the articles included in this review are illustrated in Figure 1.

## 3. Exercise Capacity and EIMD

Regular exercise has beneficial effects on health and is recommended as a useful tool to prevent disease and improve prognosis when an athlete becomes sick or injured [19]. Exercise has preventive and treatment effects on cardiovascular diseases, cancer, sarcopenia, and diabetes [37, 38]. It improves the physiological health of athletes [37]. Exercise may be linked to the gut–brain axis through resistance training and aerobic exercise [35]. An increase in oxidative stress leads to muscle damage and reduced muscle function [39]. Exercise‐induced fatigue is a common problem, and probiotics have been shown to be effective against fatigue [17]. Probiotic supplementation has been shown to increase the physical performance of athletes, swimmers, marathon runners, etc. [40, 41].

**Figure 2:**
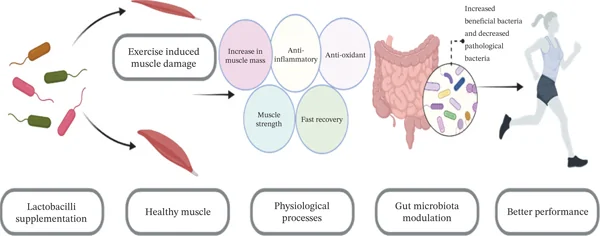
Schematic representation of the mechanisms by which *Lactobacillus* supplementation improves exercise performance. *Lactobacillus* supplementation mitigated exercise‐induced muscle damage while promoting healthy muscle maintenance through key physiological processes, including increased muscle mass, anti‐inflammatory activity and antioxidant activity, improved muscle strength, and accelerated recovery.

Various animal and human studies have shown that *Lactobacillus* strains can improve exercise capacity and prevent or reduce the severity of EIMD (Tables 1 and 2). A recent study conducted in mice revealed that *Lactobacillus casei* ATCC 27045 and *Lactobacillus paracasei* ATCC 11582, along with *Bifidobacterium* species, have antioxidant and anti‐inflammatory properties that reduce the damaging effects caused by endurance exercise [37]. *L. paracasei* PS23 isolated from healthy human feces and *Lactobacillus plantarum* PL‐02 from the feces of weightlifting women resulted in muscle damage caused by exercise, improved muscle mass and strength [4], and increased the proportion of the bacteria *Akkermansia muciniphila* [3]. Strains of *L. plantarum* taken from pickled cabbage, a frequently used fermented food, enhanced the aerobic endurance capacity. Different strains of these species, such as *L. plantarum* TWK10 and PS128, maintain exercise performance, increase the duration of endurance exercise, and alleviate muscle fatigue [35, 40, 42]. Enhancements in muscle and exercise performance, recovery from muscle damage in runners, and modification of the gut microbiota, that is, restoring decreased microbial diversity, have been reported in several studies [39, 43, 106]. Antifatigue, anti‐inflammatory, and antioxidant effects have been shown in mouse models supplemented with *Lactobacillus pentosus* CQZC02, *L. plantarum CQPC02* isolated from Sichuan pickle, *Lactobacillus rhamnosus* SDSP202418, and *Lactobacillus fermentum* CQPC08 isolated from fermented pickles, leading to recovery from muscle damage [19, 26, 27, 45, 47]. In a clinical trial, supplementation with *L. plantarum PL*‐02 and *Lactococcus lactis* subsp. lactis LY66 and a combination of *Lactobacillus acidophilus* FB0012, *L. plantarum* FB0015, *L. rhamnosus* FB0047 in soccer players increased muscle strength and performance, accelerating the recovery process of muscle damage. This was a result of reduced inflammation through gut bacteria modification and SCFA production [15, 107]. *L. paracasei* PS23 supplementation in healthy adults enhanced exercise performance [16]. Similar results were obtained with the intervention of the *plantarum* KSFY01 strain [44]. A recent study demonstrated the beneficial effect of *L. rhamnosus* GKLC1 in a mouse model on improving exercise performance through the production of SCFAs and AMPK/PGC‐1*α* signaling pathway [48]. A meta‐analysis revealed the role of different *L. plantarum* strains, TWK10, PL‐02, and *L. lactis* ly 66, in improving athletic performance through similar pathways [112]. Thus, these studies in animal models (Table 1) and human trials (Table 2) suggest that specific *Lactobacillus* strains are associated with improvement in muscle health and might be used to improve exercise performance in athletes and reduce EIMD by reducing oxidation, inflammation, and muscle protein degradation (Figure 2). This change is caused by maintenance of the gut microbiome and the production of SCFAs, which are linked to muscle health and energy.

**Table 1:** Overview of probiotic interventions targeting skeletal muscle physiology and metabolic health. The table summarizes evidence from in vitro, animal, and clinical studies, highlighting probiotic strains, experimental model′s supplementation protocols, molecular mechanisms and beneficial outcomes in exercise performance, muscle atrophy, sarcopenia, diabetes, NAFLD, and metabolic syndrome.

Bacterial strain	Experiment type	Dose and duration of supplementation	Mechanism/pathway	Outcomes	References
**Exercise performance**					
**Single strain**					
*Lactobacillus rhamnosus* SDSP202418	Kunming mice	1.0 × 10^9^ CFU/mL	Eliminates harmful serum metabolites, reduces oxidative damage, and alters muscle fiber composition. Restores gut microbial imbalance	Alleviates exercise‐induced fatigue	[27]
*Lactobacillus plantarum* TWK10	Mice	2.05 × 10^8^ CFU/kg daily for 6 weeks	Enhanced muscle mass, gastrocnemius muscle Type I fiber	Increased exercise performance	[42]
	Male ICR mice	1.03 × 10^9^ CFU kg^−1^ day^−1^ for 6 weeks	Enhances peroxisomal fatty acid oxidation. Decrease in relative abundance of Bacteroidetes and increase in Firmicutes. Decrease in *Bacteroidia*, *Rikenellaceae*, and *Alistipes*. Increase in *Clostridia*, *Porphyromonadaceae*, and *Barnesiella.*	Improves energy metabolism and exercise performance	[43]
*Lactobacillus plantarum PL*‐02	Male ICR mice	2.05 × 10^9^ CFU/kg mouse/day	Enhances glycogen storage and increases muscle mass.	Improved endurance exercise performance	[4]
	Male ICR mice	2.05 × 10^9^ CFU/kg, 4.10 × 10^9^ CFU/kg, 1.03 × 10^10^ CFU/kg, once a day for 4 weeks	Restores explosive strength and increases muscle mass	Improves endurance exercise performance	[15]
*Lactobacillus plantarum* KSFY01	Male Kunming mice	1.0 × 10^9^ CFU/kg for 4 weeks	Improves metabolite accumulation, glycogen storage, muscle and liver damage, and levels of oxidative stress.	Alleviates exercise‐induced fatigue and improves the exercise capacity	[44]
*Lactobacillus plantarum* CQPC02	ICR mice	Low dose—1.0 × 10^8^ and high dose—1.0 × 10^9^ CFU/kg	Improves skeletal muscle glycogen levels and reduces protein degradation, lactic acid accumulation, and oxidation	Antifatigue properties	[45]
*Lactobacillus fermentum* CQPC08	Kunming male mice	1.0 × 10^9^ CFU/mL	Reducing inflammatory response and oxidative stress	Alleviates exercise‐induced fatigue	[19]
*Lactobacillus salivarius* subspecies *salicinius* SA‐03	Male ICR mice	2.05 × 10^9^ CFU/kg, 4.10 × 10^9^ CFU/kg, and 1.03 × 10^10^ CFU/kg daily for 4 weeks	Increase muscle strength, endurance performance, and glycogen storage in muscle cells.	Decrease in levels of fatigue indicators and changed gut microbiota	[46]
*L. pentosus* CQZC02	Male C57BL/6J mice	Daily doses of 10^8^ CFU/kg·bw and 10^9^ CFU/kg·bw for the low and high concentration groups, respectively, over a 30‐day period	Mitigate oxidative inflammation	Improvement in exercise performance	[47]
*L. rhamnosus* GKLC1	Male ICR mice	0.021 g/kg/day for 6 weeks	AMPK/PGC‐1*α* signaling pathway, mitochondrial biogenesis, and glycogen homeostasis	Enhanced endurance performance	[48]
**Multistrain**					
*L. casei* ATCC 27045, *L. paracasei* ATCC 11582, *B. longum* DSM‐20219, *B. bifidum* ATCC 29521, and *B. breve* ATCC 15700	Male C57BL/6 mice	Total cell density of 1 × 10^10^ cells/mL for 4 weeks	Activation of antioxidant enzymes, reduction of inflammatory markers, and enhancement of mitochondrial biogenesis	Improves exercise endurance	[37]
**Muscle atrophy**					
**Single strain**					
*Lactobacillus rhamnosus* JY02	Mouse myoblast C2C12 skeletal muscle cells	1 × 10^8^ CFU/mouse for 5 weeks	Reduces atrophic muscle factors and pro‐inflammatory cytokines. Produces muscle differentiation markers.	Ameliorates muscle atrophy	[49]
*Lactobacillus curvatus* CP2998	C2C12 myoblast cells	Cells treated with heat killed *L. curvatus* for 24 h	Suppression of glucocorticoid receptor‐ dependent transcription	Prevents dexamethanose‐induced muscle atrophy	[1]
*Lactobacillus curvatus* HY7602	C2C12 myoblasts, male Hsd:ICR mice	Fermented Antler (120 mg/kg body weight) for 24 days	Inhibits muscle protein breakdown, recovers muscle loss, and promotes muscle protein synthesis	Mitigates muscle atrophy	[13]
	Male BALB/c mice	Fermented Antler once a day for 4 weeks	Inhibits muscle protein breakdown and apoptosis. Enhances muscle fiber regeneration, recovers muscle loss, and promotes mitochondrial biogenesis	Inhibits sarcopenia	[6]
*Lactobacillus paracasei* PS23	C57BL/6J mice	10^9^ CFU/day/mice daily for 9 weeks	Enhances the activation of Akt, maintains ghrelin levels, and increases muscle strength.	Ameliorates muscle atrophy	[12]
	SAMP8 mice	Omega‐3 fatty acid (500 mg), leucine (2.5 g), probiotic LPPS23 (“30 Billion,” freeze dried), once a day	Decreases inflammatory markers and ROS and maintains mitochondrial function	Delays the progression of sarcopenia	[50]
	SAMP8 mice	1 × 10^9^ CFU/mouse/day	Attenuated decrease in muscle loss and strength and increased mitochondrial function, IL10, antioxidant enzymes, and protein uptake. With a decrease in inflammatory cytokines and ROS	Attenuated sarcopenia progression	[51]
*Lactobacillus plantarum* TWK10	Male ICR mice	1 × 10^9^ CFU/mouse/day for 8 weeks	Improves muscle quality and muscle glycogen levels, reduces the accumulation of pathogens, and enhances SCFA producing bacteria levels.	Mitigates muscle weakness	[52]
*Lactobacillus plantarum* HY7715	Aged BALB/c mice and C2C12 cells	1 × 10^9^ CFU/kg/day	Reduced levels of TNF *α*, enhanced muscle function, and elevated levels of MyoD and myogenin. Enhanced mitochondrial biogenesis and altered gut microbiota	Ameliorates sarcopenia	[3]
*L. plantarum* beLP1	C2C12 myotubes and male Sprague–Dawley rats	In vitro: 50, 100, and 250 *μ*g/mL for 48 h in vivo: 1 and 3 mg/kg administered once daily for 19 days	Regulates the AKT/FoxO3*α* signaling pathway, increases AKT phosphorylation reducing the expression of FoxO3*α*, and inhibits the (UPS) by downregulating the expression of MuRF1 and Atrogin‐1	Ameliorates sarcopenia	[53]
*Lactobacillus casei* Shirota	SAMP8 mice	Low‐dose 1 × 10^8^ CFU LcS/mouse/day and high‐dose 1 × 10^9^ CFU LcS/mouse/day for 12 weeks	Reduce inflammation and ROS, maintain SCFA (butyric acid) levels, and regulate gut microbiota composition	Attenuates sarcopenia	[54]
*Lactobacillus casei* LC122	Male C57BL/6 mice	12 weeks at a dose of 2 × 10^9^ CFU mouse^−1^ day^−1.^	Improved muscle function, decreased inflammation and oxidative stress, and altered gut microbiota	Improved muscle function and anti‐aging properties	[24]
*L. gasseri* IM13	C2C12 cell line	Whey protein administered to the cells for 48 h	Activated the phosphorylation of the IGF‐1/PI3K/AKT pathway and enhanced muscle homeostasis. Dephosphorylated FOXO3a and increased muscle protein synthesis. Increased myogenesis and antioxidant activity	Prevents muscle atrophy	[55]
*L. gasseri*	C2C12 myotubes and LPS‐stimulated RAW264.7 macrophages, male C57BL/6J mice	1 × 10^8^ CFU/kg/day administered daily for a total of 3 weeks	Maintains FOXO3a phosphorylation, suppresses the expression of the E3 ubiquitin ligases Atrogin‐1 and MuRF1, and promotes myogenesis by restoring myogenin and MyHC isoforms along with anti‐oxidant and anti‐inflammatory properties	Ameliorates muscle atrophy	[55]
*L. fermentum* LF31	Female BALB/c mice.	10^9^ CFU per animal for 8–12 weeks	Mitigates oxidative stress by reducing Hsp60 and reduces inflammation and enhances muscle regeneration	Mitigates muscle atrophy	[56]
*L. plantarum* MIRE_TS55	C2C12 myoblasts and male C57BL/6 mice	In vivo—1000 or 2000 mg/kg administered orally for a total of 14 weeks. In vitro—50, 100, 200, and 400 *μ*g/mL for 24 h	Akt/mTOR/FoxO signaling pathway, increase in branched chain amino acids, PGC‐1*α* for mitochondrial function and suppressing atrophy markers myostatin, atrogin‐1, and MuRF1	Mitigates muscle weakness	[57]
**Multistrain**					
*Lactobacillus paracasei* P62 and *Bifidobacterium bifidum* P61	Aged C57BL/6 male mice, C2C12 cells	1 × 10^9^ CFU/mouse of each species, once a day for 8 weeks	Regulates gut microbiota‐mediated AKT, NF‐*κ*B, and/or FOXO3a signaling pathways	Ameliorates muscle atrophy and weight loss	[5]
**Diabetes**					
**Single strain**					
*L. rhamnosus* GG	Male Wistar rats	10^9^ CFU per day	Higher CPT1‐b activity and improved GLUT‐4 expression in the skeletal muscle.	Prevents insulin sensitive reduction	[58]
	Male C57BL/KsJ mice	1 × 10^8^ CFU per mouse daily for 4 weeks	ER stress in skeletal muscle alleviated and activation of M1‐like macrophage	Antidiabetic properties	[59]
*Lactobacillus rhamnosus* ZJUIDS07	Male C57BL/6 mice	10^9^ CFU/mL	Improved glucose mechanism and insulin sensitivity and reduced inflammatory markers. Alleviates oxidative stress. Restoration of gut microbes. Increase—*Muribaculaceae_norank*, *Dubosiella*, *Clostridium sensu stricto 1*, *Lactobacillus*, *Faecalibaculum*, *Blautia*, and *Roseburia.* Decrease—*Staphylococcus*, *Corynebacte rium*, *Odoribacter*, *Coriobacteriaceae*_*UCG-002*, and *Alistipes*. Increased SCFA such as acetic, propionic, and butyric acids	Ameliorates Type 2 diabetes	[60]
*Lactobacillus paracasei* IMC 502	Male C57BL/6J mice	10^9^ CFU day^−1^	Alters gut microbiota and SCFA (acetic/butyric acid) levels and decreases blood sugar and HbA1c levels. Repairs glucose intolerance, lipid metabolism, and insulin sensitivity.	Antidiabetic	[61]
*Lactobacillus plantarum* HAC01	C67BL/6J mice	1 × 10^9^ and 4 × 10^9^ CFU day^−1^ for 10 weeks	Decrease in blood glucose and HbA1c levels. Improved glucose tolerance, activation of the Akt and AMPK pathways, and shift in gut microbiota	Ameliorated T2D	[28]
*L. fermentum* ME‐3	db/db mice	10^10^ CFU per 400‐*μ*L H_2_O over 12 weeks.	Improved glucose tolerance and reduced glycation	Antidiabetic properties	[62]
*L. reuteri* TISTR 2736	Male Sprague–Dawley rats	2 × 10^8^ CFU/day for 30 days	Upregulation of PI3K and phosphorylated AKT. Suppressed oxidative stress and inflammation	Alleviates T2D	[63]
*Lactobacillus casei* NCDC‐017	Male Wistar rats	1 × 10^7^ cfu/mL suspended in 1 mL of distilled water for 28 days	Ameliorated hyperglycemia, dyslipidemia, and oxidative stress	Antidiabetic properties	[64]
**Multistrain**					
*Lactobacillus acidophilus* KLDS1.1003 and KLDS1.0901	Male C57BL/6J mice	10^8^ cfu/mL for 8 weeks	Inhibit inflammatory responses, regulate lipid and glucose metabolism, increased relative abundance of Bacteroidetes, increased levels of *Bifidobacterium*, *Lactobacillus*, *Alloprevotella*, and SCFA producing bacteria—*Blautia*, *Roseburia*, and *Anaerotruncus.* Decreased levels of *Desulfovibrio*, *Bacteroides*, and *Alistipes.* Increase in SCFA such as butyric acid.	Alleviates T2D	[65]
*L. plantarum* Lp91 and *L. fermentum* Lf1	Male C57BL/6J mice	1.5 × 10^9^ colonies/mouse/day	Improved insulin sensitivity and enhanced glucose tolerance and resistance to the occurrence of diabetes. Increased levels of GLP‐1, restored ER stress markers, decrease in inflammation, lipogenesis, and gluconeogenesis	Mitigates diabetes and obesity	[66]
*L. rhamnosus* BSL and *L. rhamnosus* R23	Male Sprague–Dawley rats	10^9^ CFU/mL daily for 30 days	Decreased blood glucose, inflammation, and decreased pathogenic bacteria	Ameliorates T2DM	[67]
*Lactobacillus fermentum* MCC2759 and MCC2760	Female Wistar rats	10^9^ CFU/mL suspended in PBS, once a day for 4 weeks	Pro‐inflammatory cytokines in muscle tissue reduced	Antidiabetic properties	[68]
**Obesity**					
**Single strain**					
*L. rhamnosus* GG	Male C57BL/6 mice	1 × 10^7^ CFU/day for 10 weeks	Weight and fat mass reduced, microbiota restored, and increased levels of SCFA	Anti‐obesity effects	[69]
*Lactobacillus plantarum* HT121	Male C57BL/6n mice	3 × 10^8^ CFU/mL for 11 weeks	Reduction in weight gain, improved glucose tolerance, SCFA levels increased, and restored gut bacteria	Prevents obesity	[70]
*L. plantarum* dy‐1	T3‐L1 cell line	Cells were exposed to 50 *μ*g/mL LFBE, 100 *μ*g/mL LFBE, and 100 *μ*g/mL LFBE	Decrease in inflammation and oxidative stress with gut microbiota modulation	Anti‐obese properties	[71]
*Lactobacillus plantarum* L‐137	Male C57/BL6J mice	0.002% heat killed bacteria for 4–20 weeks	Prevents weight gain and inflammation	Attenuates obesity	[72]
*Lactobacillus plantarum* KC28	3 T3‐L1 cells	Cells treated with 100 *μ*g/mL of *L. plantarum* KC28 (10^9^ CFU/mL)	Upregulation of PGC1‐*α* and CPT1‐*α*, downregulation of ACOX‐1 and PPAR‐*γ* and increase in the families *Desulfovibrionaceae* and *Lactobacillaceae*	Attenuates obesity	[73]
*L. casei* Zhang	C57BL/6J mice	4 × 10^9^ CFU live bacteria daily	Increased levels of *Enterorhabdus mucosicola* decreased *Lactobacillus reuteri.* Reduction of body weight and alteration of biosynthetic and metabolic pathways	Ameliorates obesity	[74]
*Lactobacillus acidophilus* NS1	Male C57BL/6 mice	10^8^ cfu/mL for 8 weeks	Alters lipid metabolism through the AMPK‐PPAR*α*/SREBP‐1c pathway and attenuates insulin resistance	Ameliorates obesity	[75]
*Lactobacillus kefiri* DH5	Male C57BL/6J mice	2 × 10^8^ cfu	Reduced cholesterol and upregulated the expression of PPAR‐*α*	Anti‐obese properties	[76]
*Lactobacillus acidophilus* JYLA‐126	Male C57BL/6 mice	5 × 10^9^ CFU/mL	Restores gut microbial imbalance by enhancing the relative abundance of Bacteroidetes, decreasing Firmicutes and Proteobacteria, reducing inflammation, and activating AMPK signaling pathway	Inhibits obesity	[77]
*L. acidophilus* YL01	SPF C57BL/6J male mice	10^8^ CFU/mL everyday	Altered gut microbiota and increased SCFA levels. Activation of AMPK and ACC signaling pathways and decreased inflammation	Ameliorates obesity and insulin resistance.	[78]
*L. buchneri* HMPM2112	Male C57BL/6 mice	2 × 10^9^ CFU/head/2 days	Improved hepatic lipid profiles, reduced body weight, and altered gut microbiota.	Anti‐obese properties	[26]
*Lactobacillus paragasseri* SBT2055	Sprague–Dawley rats	6 × 10^7^ colony‐forming unit/g diet.	Reduces inflammation and increases SCFA levels	Anti‐obesity properties	[79]
*L. delbrueckii* subsp. *lactis* CKDB001	Male C57BL/6J mice	1.0 × 10^9^ CFU per mouse for 12 weeks	Enhanced AMPK phosphorylation and regulated energy metabolism	Ameliorates obesity	[80]
**Multistrain**					
*L. rhamnosus* 069 and *L. brevis* 031	Male C57BL/6 mice	5 × 10^8^ CFU/mL/day	Enhances lipid metabolism, reduces inflammatory markers, regulates gut microbiota, and enhances the production of SCFA	Attenuates obesity	[23]
*L. plantarum* L14, *L. paracasei* L9, *L. rhamnosus* GG, and *L. sakei* X‐MRS‐2	Male C57BL/6 mice	1 × 10^9^ CFU/mL for 6 or 12 weeks	Decrease in inflammatory markers such as TNF‐*α*, IL‐1*β*, and IL‐6. Elevated *Lactobacillus*, *Akkermansia*, *Dubosiella*, and *Muribaculaceae* and reduced *Helicobacter* levels.	Anti‐obese properties	[81]
*Lacticaseibacillus rhamnosus* MG4502, *Lactobacillus gasseri* MG4524, *Limosilacto bacillus reuteri* MG5149, and *Weissella cibaria* MG5285	Male C57BL/6J mice	2 × 10^8^ CFU/mouse	Prevented body weight gain, improved glucose tolerance and lipid metabolism.	Anti‐obese properties	[53]
*Lactobacillus paracasei* TD3 and *B. coagulans* T4	C57BL/6J mice	1 × 10^9^ CFU/day	Suppresses macrophage infiltration and shifts the phenotype from pro‐inflammatory to anti‐inflammatory. Reduction in pro‐inflammatory cytokines and enhancement of antioxidants	Partial prevention of obesity‐induced inflammation	[29]
**NAFLD and metabolic syndrome**					
**Single strain**					
*L. rhamnosus* GG	Male C57BL/6J mice	Daily intake of 1 × 10^8^ CFU	Regulates inflammation and oxidation	Anti‐NAFLD properties	[82]
	Male Wistar rats	1 × 10^9^ CFU/day	Antioxidative and anti‐inflammatory activities	Anti‐NAFLD properties	[58]
	Male C57BL/6J mice	1 × 10^8^ CFU per mouse daily for 13 weeks	Reduction of pro‐inflammatory cytokines and enhancement of bile acids	Protects against NAFLD	[57]
*L. rhamnosus* hsryfm 1301	Male Wistar rats	1 × 10^9^ cfu/100 g BW	Reduction in reactive oxygen species and free fatty acids. Ameliorated inflammation	Anti‐NAFLD effects	[83]
*L. plantarum* ATCC14917	Male Sprague–Dawley rats	1 × 10^9^ CFU/ml	Reduced oxidative stress and inflammation and partly modulated the gut microbiome	Mitigate NAFLD	[84]
*L. plantarum* S9	Healthy male Sprague–Dawley (SD) rats	2 mL once daily for 6 weeks	Reduced weight gain, regulated lipid metabolism disorder and insulin resistance, and suppressed TLR4/NF‐*κ*B pathway and inflammation.	Ameliorates metabolic disorder	[85]
*L. plantarum* Q180	Male C57BL/6 mice	Low‐dose (1 × 10^9^ CFU/day) and high‐dose (1 × 10^10^ CFU/day)	Reduces adiposity and improves lipid and glucose intolerance. Alters gut microbiota and upregulated irisin in skeletal muscle	Treatment of metabolic disorders	[86]
*L. plantarum ZJUIDS14*	Male C57BL/6 mice	10^9^ CFU/every other day for 12 weeks	Altered gut microbiota, improved mitochondrial function, and increased fatty acid oxidation.	Alleviates NAFLD	[87]
*Lactobacillus plantarum* MA2	Rats	10^9^ CFU per mL	Reduced the growth of harmful bacteria and regulated the activation of TLR‐4	Alleviates NAFLD	[88]
*Lactobacillus plantarum* NA136	Male C57/BL6J mice	1.0 × 10^9^ CFU/day/mouse for 16 weeks	Modulating gut microbiota, improving intestinal barrier integrity, and attenuating inflammation	Mitigates NAFLD	[89]
*L. plantarum* K6 and K2	Male Wistar rats	5 × 10^6^ CFU	Regulated antioxidant enzymes and liver function	Ameliorated NAFLD	[90]
*L. fermentum* CQPC06	Male C57/BL6J mice	1.0 × 10^9^ CFU kg^−1^	Increased gut microbial diversity and levels of beneficial bacteria	Prevents NAFLD	[91]
*L. fermentum* CRL1446	Male Swiss albino mice	100 *μ*L of *L. fermentum* CRL1446 suspension	Enhanced oxidative status, modulation of gut microbiota, improved inflammatory profile, and reduced weight	Improved metabolic status	[92]
*Lactobacillus brevis* SBL88	Male C57BL/6J mice and Huh 7 cells	1% heat‐killed *L. brevis* SBL88 for 16 weeks	Improves insulin resistance	Attenuates NAFLD	[93]
*Lactobacillus acidophilus* KLDS1.0901	Hepatoma G2 cells and male C57BL/6J mice	5 × 10^9^ CFU mL^−1^	Reduced inflammation and modulated tryptophan levels	Mitigates NAFLD	[94]
*Lactobacillus reuteri* FYNLJ109L1	Male C57BL/6J mice	0.2 mL of freeze dried powder	Reduced weight gain, fat accumulation, insulin resistance, and dyslipidemia. Decrease in *Romboutsia* and *Clostridium sensu stricto 1* and an increase in *Acetatifactor*	Alleviates metabolic syndrome	[95]
*Lactobacillus reuteri* MJM60668	HepG2 cells	10^8^–10^9^ CFU/mL	Inhibits inflammation, improves gut microbial dysbiosis, and inhibits adipogenesis.	Prevents the progression of NAFLD	[96]
*Lactobacillus coryniformis* supsp. *torquens* T3	Male BALB/c mice	2 mL per 100 g body weight, for 6 weeks	Restores gut microbiota and inflammatory cytokine levels	Attenuates NAFLD and obesity	[97]
*Lactobacillus johnsonii* JNU3402	Male C57BL/6J mice	Two‐hundred microliters of bacterial resuspension daily for 14 weeks	Improves obesity and insulin resistance	Attenuates NAFLD	[98]
*L. reuteri* ZJ617	Male C57BL/6J mice	10^9^ colony‐forming units/mL	Production of sperimidine and prevents the decline of bacteria	Ameliorated symptoms of metabolic syndrome	[99]
**Multistrain**					
*L. paracasei* CNCM I‐4270 and *L. rhamnosus* I‐3690	Male C57BL/6J mice	0.2 mL of freeze‐dried powder	Mitigates obesity and inflammation. Increase in *Bifido bacterium*, *Olsenella*, *Barnesiella*, *Allobaculum*, *Butyrivibrio*, unclassified *Lachnospiraceae* and unclassified *Proteobacteria* levels. Decrease in Alistipes, *Desulfovibrionaceae*, *Oscillibacter*, *Clostridium* XIVb, and *Dorea* and *Clostridium* XIVa levels.	Alleviates metabolic syndrome	[100]
*L. rhamnosus* FJSYC4‐1 and *L. reuteri* FGSZY33L6	Male C57BL/6J mice	0.2 mL of bacterial solution, 6 times a week for 12 weeks	Reduction in blood glucose and insulin levels and increased levels of butyric acid (SCFA). Enhanced levels of *Lachnospiraceae_UCG_006* and *Ruminiclostridium.* Decrease in *Romboutsia*	Ameliorates metabolic syndrome	[101]
*L. pentosus* GSSK2, *L. fermentum* PUM, and *L. plantarum* GS26A	Male Sprague–Dawley rats	Single dose of 0.1 mL containing 1 × 10^9^ lactobacilli for 12 weeks	Regulated insulin resistance, energy metabolism, and plasma glucose.	Attenuates metabolic syndrome	[102]
*L. acidophilus* BCMC 12130, *L. casei* subsp. BCMC 12313, *L. lactis* BCMC 12451, *Bifidobacterium bifidum* BCMC 02290, *Bifidobacterium infantis* BCMC 02129, and *Bifidobacterium longum* BCMC 02120	NAFLD patients	*L. acidophilus* BCMC 12130 (107 mg), *L. casei* subsp. BCMC 12313 (107 mg), *L. lactis* BCMC 12451 (107 mg), *Bifidobacterium bifidum* BCMC 02290 (107 mg), *Bifidobacterium infantis* BCMC 02129 (107 mg), and *Bifidobacterium longum* BCMC 02120 (107 mg). 3‐g sachet twice a day for 6 months.	Decreased pro‐ inflammatory cytokines and level of pathogenic gut microbes	Anti‐NAFLD properties	[103]
*Lactobacillus paracasei* CNCMI‐4034, *Lactobacillus rhamnosus* CNCM I‐4036, and *Bifidobacterium breve* CNCM I‐4035	Zucker‐Lepr male rats	10^10^ CFUs for 30 days	Improved liver damage and peroxidation	Anti‐NAFLD effects	[104]
*Lactiplantibacillus plantarum* LP158, *Lactobacillus helveticus* HY7804, and *Lacticaseibacillus paracasei* LPC226	Human hepatoma cells	Each strain 10^9^ CFU/kg/day	Inhibits inflammation and fibrosis	Ameliorates NAFLD	[49]
**Combination with other ingredients**					
*L. paracasei* N1115 and fructooligosaccharides	C57BL/6J mice	2.2 × 10^9^ CFU/mL in normal saline, 0.5 mL/day	Reduces inflammation and overcomes insulin resistance	Prevents NAFLD	[105]

**Table 2:** Summary of human clinical studies investigating the effects of probiotic supplementation of skeletal muscle function and muscle‐associated metabolic disorders in humans. The table summarizes the probiotic strain (s), study population, supplementation dose and duration, proposed molecular mechanism or signaling pathways, and clinical outcome.

Bacterial strain	Experiment type	Dose and duration of supplementation	Mechanism/pathway	Outcomes	Author, year
**Exercise performance**					
*Lactobacillus paracasei* PS23	Healthy adults	1 × 10^10^ cells/capsule, 2 capsules/day	Reduced muscle force loss, blood biomarkers of muscle injury, and inflammation caused due to muscle damage	Enhances exercise performance	[16]
*Lactobacillus plantarum* PS128	Triathlon subjects	Each capsule included 300 mg of lyophilized bacteria powder, twice a day.	Change in inflammation, oxidation and metabolic markers	Elevates exercise performance	[40]
	Triathlon participants	Each capsule included 400 mg of lyophilized bacteria powder, twice a day	Alters gut microbial community and ameliorates oxidative stress and inflammation	Improves high‐intensity exercise performance	[106]
	Recreational runners	Each capsule contains 400 mg of lyophilized bacterial powder, which contains 300 mg of lyophilized PS128 powder, twice a day	Increases strength	Mitigates muscle fatigue	[35]
	Recreational runners	2 capsules (3 10^10^ CFU/capsule) for 2 weeks	Improvement in muscle damage	Regulates immune response and oxidative stress	[39]
*Lactobacillus plantarum PL*‐02 and *Lactococcus lactis* subsp. Lactis LY66	Healthy adults	7.5 × 10^9^ CFU/sachet, 2 sachets/day in 250 mL of water	Increase in *Lactobacillus*, *Akkermansia muciniphila*, and *Lachnospiraceae* and reduction in *Sutterella*. Increase in SCFA levels and decreased IL‐6 levels.	Enhances muscle strength and endurance performance	[15]
*L. acidophilus* FB0012, *L. plantarum* FB0015, and *L. rhamnosus* FB0047	Male elite soccer players and healthy volunteers	Each capsule with 10–35 billion cfu of the strains. One capsule per day for 14 days.	Changes in oxidative stress markers, BAP, D‐ROMS, cortisol, inflammatory markers, TNF‐*α*, IL‐6, and modulation of *Faecalibacterium prausnitzii* and *B. longum* in gut	Increase in energy in athletes	[107]
*Lactobacillus plantarum* TWK10	Healthy participants without athletic training	Low dose (3 × 10^10^ CFU/day) and high dose (9 × 10^10^ CFU/day)	Regulation of energy balance and metabolism	Elevated endurance exercise capacity	[40]
**Combination with other ingredients**					
*Lactiplantibacillu splantarum* BP06, *Lacticaseibacillus casei* BP07, *Lactobacillus acidophilus* BA05, *Lactobacillus bulgaricus* BD08, *Bifidobacterium infantis* BI04, *Bifidobacterium longum* BL03, *Bifidobacterium breve* BB02, and *Streptococcus thermophilus* BT01 and omega‐3	Male sprint swimmers	*Lactiplantibacillu splantarum* BP06 0.43 × 10^11^ *Lacticaseibacillus casei* BP07 0.65 × 10^11^ *Lactobacillus acidophilus* BA05 0.94 × 10^11^ *Lactobacillus bulgaricus* BD08 0.36 × 10^11^ *Bifidobacterium infantis* BI04 0.57 × 10^11^ *Bifidobacterium longum* BL03 0.73 × 10^11^ *Bifidobacterium breve* BB02 0.62 × 10^11^ *Streptococcus thermophilus* BT01 0.20 × 10^11^ Total 4.5 × 10^11^ CFU, in a capsule and omega‐3 capsule, once a day	Enhanced both in‐water and dry‐land performance	Improved swimming performance	[41]
**Muscle atrophy**					
*Lactobacillus plantarum* TWK10	Elderly subjects	Capsule containing 1 × 10^10^ or 3 × 10^10^ CFU	Improved hand grip strength and increased muscle mass	Improves muscle health	[52]
**Diabetes**					
*L. reuteri* ADR‐1 or ADR‐3	T2D patients	2 × 10^9^ CFU/capsule for ADR‐1, 1 × 10^10^ cells/capsule for or ADR‐3	Significant reductions in HbA1c and modification of intestinal flora	Anti‐ T2D effects	[108]
**Obesity**					
*Lactobacillus plantarum PL*‐02 and *Lactococcus lactis* subsp. lactis	Healthy adults	7.5 × 10^9^ CFU/sachet, 2 sachets/day in 250 mL of water	Increase in *Lactobacillus*, *Akkermansia muciniphila*, and *Lachnospiraceae* and reduction in *Sutterella*. Increase in SCFA levels and decreased IL‐6 levels.	Enhances muscle strength and endurance performance	[15]
*Lactobacillus plantarum* LMT1‐48	Human subjects	LMT1‐48 placebo	Modulation of gut microbiome	Prevents obesity	[109]
*L. sakei* CJLS03	Human subjects	Daily two allocations of 5 × 10^9^ CFU for 12 weeks	Reduction in fat mass and body weight	Anti‐obesity effects	[110]
**Metabolic syndrome**					
*L. reuteri* V3401	Metabolic syndrome patients	5 × 10^9^ CFU capsules, once a day for 12 weeks	Improved inflammatory markers and enhanced *Verrucomicrobia*	Improves metabolic syndrome condition	[111]

## 4. Metabolic Disorders

**Figure 3:**
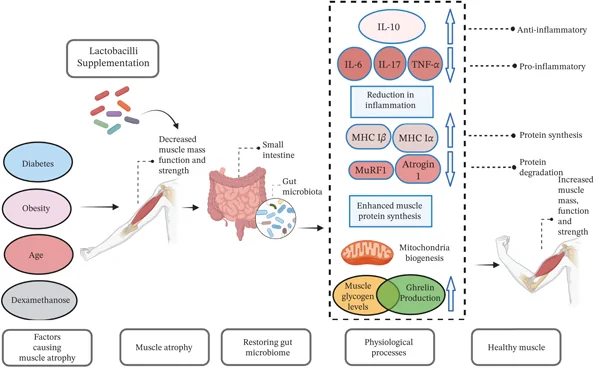
Schematic representation of the mechanism by which *Lactobacillus* supplementation attenuates muscle atrophy induced by diabetes, obesity, aging, and dexamethasone treatment. *Lactobacillus* supplementation restored gut microbiota homeostasis, which in turn drove multiple physiological adaptations, including a reduction in pro‐inflammatory cytokines (IL‐6, IL‐17, and TNF‐*α*) and an increase in anti‐inflammatory markers (IL‐10), enhanced muscle protein synthesis via the upregulation of MHCI*β* and MHC I*α* with the concurrent suppression of the proteolytic markers MuRF1 and Atrogin‐1, the promotion of mitochondrial biogenesis, improved muscle glycogen storage, and the modulation of ghrelin production.

As mentioned earlier, muscle atrophy is related to metabolic disorders such as obesity and diabetes and metabolic syndromes, including NAFLD [7, 9, 11, 113]. The risk and severity of muscle loss are greater in patients with metabolic disorders [114]. Hence, reducing the risk or curing these diseases might reduce the incidence of muscle atrophy. The administration of *Lactobacillus* has shown promising results in attenuating these metabolic disorders (Figure 3 and Figure S1).

### 4.1. Diabetes Mellitus

Diabetes mellitus is the third most common noncommunicable endocrine system disease and is characterized by abnormally high blood glucose levels due to impaired insulin secretion or insulin resistance [60]. Muscle loss or sarcopenia and diabetes are related through mechanisms such as oxidative stress and insulin resistance and are termed diabetic sarcopenia [114]. *L. acidophilus* KLDS1.1003 and KLDS1.0901 supplementation in high‐fat diet‐fed mice reduced inflammation, prevented insulin resistance, and maintained lipid homeostasis by increasing microbial richness and diversity, that is, decreasing the number of pathogenic, stress‐tolerant microbes [65]. *L. plantarum* HT121 enhanced glucose tolerance, restored the Firmicutes/Bacteroidetes (F/B) ratio, restored the abundance of beneficial bacteria, and hence reversed the gut microbial structure. While alterations in the F/B ratio have been associated with metabolic dysfunction in numerous studies, the interpretation of this ratio remains a subject of debate in the microbiome field [70]. In diabetic mice, other strains of *Lactobacillus*, including *L. plantarum* HAC01, *L. rhamnosus* BSL, and *L. rhamnosus* R23, have antihyperglycemic effects, maintain glucose levels, increase SCFA levels, including butyric acid levels, and restore the gut microbial composition [28, 60, 67]. The intervention *of L. rhamnosus* ZJUIDS07 isolated from traditionally fermented dairy products in murine diabetic models restored the gut microbiota. This was achieved by increasing SCFA‐producing beneficial bacterial species and reducing the growth of harmful gut bacteria associated with insulin resistance and intestinal inflammation. The resulting SCFAs influence the quality and activity of pancreatic beta cells by reducing the levels of proinflammatory cytokines, including TNF‐*α*, a driver of insulin resistance, while increasing the levels of anti‐inflammatory cytokines such as IL‐10. Probiotic intervention also increased the activity of antioxidant enzymes such as SOD and GSH‐Px and reduced the expression of the oxidative stress marker MDA, leading to a decrease in ROS levels. Together, these changes protected pancreatic *β*‐cells from dysfunction and apoptosis. The intervention further restored the conversion of glucose into hepatic glycogen and normalized lipid profiles. These changes resulted in a significant reduction in fasting blood glucose and glycated hemoglobin (HbA1c) levels, along with improved insulin sensitivity, thereby ameliorating T2D [60]. *Lactobacillus reuteri* ADR‐1 or ADR‐3 supplementation in human diabetic patients increased the level of gut bacteria and reduced HbA1c levels, with positive results [108]. *L. paracasei* IMC 502, *L. reuteri* TISTR 2736, *L. fermentum* ME‐3, and *L. casei* NCDC‐017 supplementation attenuated diabetes and related processes in high‐fat diet‐ and STZ‐induced animal models in different studies [61–64]. These findings suggest the role of SCFAs as a link between probiotic supplementation and antidiabetic effects. Probiotic supplementation promotes the growth of beneficial gut bacteria, leading to increased SCFA production. These metabolites act on pancreatic *β*‐cells, inflammatory cytokines, and oxidative markers, improving metabolism and contributing to desirable outcomes. However, the underlying molecular pathway remains unclear. Most of the available evidence for the antidiabetic effects of *Lactobacillus* comes from experimental models (Table 1), whereas human studies (Table 2) remain limited. Preclinical findings suggest that selected strains can improve glucose homeostasis, reduce inflammation, and modulate the composition of the gut microbiota. However, human clinical evidence is still insufficient to confirm these effects for routine therapeutic use. These findings highlight the influence of certain *Lactobacillus* strains on improving diabetic conditions and suggest that their combination with other probiotic strains might have a better effect on human health.

### 4.2. Obesity

Obesity is a progressive noncommunicable disease characterized by the accumulation of excess body fat. Sarcopenic obesity is characterized by a decrease in lean body mass and excessive accumulation of adipose tissue, and the common mechanism that links obesity and muscle loss is the proinflammatory state. The *L. rhamnosus* 069 and *L. brevis* 031 strains increase acetic acid levels and alter the gut microbiota composition and functionality by reducing the F/B ratio and increasing beneficial SCFA production, along with anti‐obesity effects [23]. Similar results have been obtained for *Lactobacillus* species isolated from fermented foods in animal models. The supplementation of *L. plantarum* KC28 isolated from kimchi, a traditional fermented Korean food, in obesity‐induced mouse models resulted in significant improvement in SCFAs, primarily butyrate produced by gut microbes, upregulated the genes involved in the burning of fat, such as PGC1‐*α*, CPT1‐*α*, and PPAR‐*α*, and downregulated the expression of FAS, PPAR‐*γ*, and ACOX‐1, which are indicators of fatty acid synthesis and accumulation in mesenteric adipose tissue [73]. *Lactobacillus buchneri* HMPM2112 isolated from pickled garlic and *L. acidophilus* NS1 demonstrated the same pathway [26, 75]. *L. acidophilus* NS1 strains in high‐fat diet‐fed mice and HepG2 cells, *L. casei* Zhang, *Lactobacillus paragasseri* SBT2055, and *Lactobacillus kefiri* DH5 exhibit AMPK and PPAR*α* signaling pathway, leading to improved insulin sensitivity due to the increase in Akt phosphorylation in the liver, skeletal muscle, and white adipose tissue [74–76, 79]. Restoration of the gut microbiota and regulation through AMPK signaling were observed in the *L. acidophilus* JYLA‐126 and *Lactobacillus delbrueckii* subsp. *lactis* CKDB001 strains [77, 80]. The *L. acidophilus* strain isolated from fermented dairy food has been shown to prevent metabolic disorders such as diabetes and obesity [11, 65, 69, 73]. In vitro studies with the *L. plantarum* dy‐1 strain and clinical trials with the *L. plantarum* LMT1‐48 strain have also shown the anti‐obesity properties of *L. plantarum* [71, 109]. In a clinical trial, the *Lactobacillus sakei* CJLS03 strain produced positive results, suggesting that supplementation might help improve the health of obese individuals [110]. Together, the *L. plantarum* Lp91 and *L. fermentum* Lf1 strains improved diabetes and obesity in mice by increasing insulin sensitivity and glucose tolerance and GLP‐1 levels [66]. Collectively, the available results suggest a common molecular pathway, that is, the production of SCFAs that downregulate genes involved in fatty acid synthesis, such as FAS, PPAR‐*γ*, ACOX‐1, and upregulate those involved in fat burning, such as PGC1‐*α*, CPT1‐*α*, and PPAR‐*α*. The regulation of these genes promotes Akt phosphorylation, reduces inflammation, and improves metabolic health. These results, shown in preclinical (Table 1) and clinical trials (Table 2), indicate that the adverse effects of obesity and diabetes can be minimized by supplementation with probiotics consisting of *Lactobacillus* strains (Tables 1 and 2). Further clinical trials can better determine whether supplementation can be helpful in improving these disease conditions.

### 4.3. Metabolic Syndrome and NAFLD

Different strains of *L. acidophilus* attenuate NAFLD in different models; for example, *L. acidophilus* KLDS1.0901 supplementation in animal models reduced inflammation and modulated tryptophan levels [94, 115] (Tables 1 and 2). Yogurt fermented with *L. acidophilus* La5 with *Bifidobacterium lactis* Bb12 improved insulin sensitivity and reduced oxidative stress, thereby ameliorating NAFLD and metabolic syndrome [116]. *L. plantarum* S9 reduces the expression of proinflammatory cytokines and ameliorates glucose metabolism disorders [85]. *L. pentosus* GSSK2, *L. fermentum* PUM, and *L. plantarum* GS26A attenuate metabolic syndrome in rat models [102]. *L. reuteri* FYNLJ109L1 decreased insulin resistance, dyslipidemia, and fat accumulation, *L. paracasei* CNCM I‐4270 and *L. rhamnosus* I‐3690 attenuated obesity and inflammation in mice with metabolic syndrome via modulation of the gut microbiota [95, 100, 117]. These findings are consistent in an animal model in which two strains, *L. paracasei* CNCM I‐4270 and *L. rhamnosus* I‐3690, were administered for the amelioration of metabolic syndrome [100]. In vivo studies have shown that *L. paracasei* N1115 is a promising probiotic species for preventing NAFLD [54, 105, 118, 119]. Another common species of *Lactobacillus* that has been used in several studies is *L. plantarum* ATCC14917, ZJUIDS14, MA2, NA136, K2, and K6. Its administration improved oxidative stress [90], reduced inflammation [89], modified the gut microbial community [84, 87, 88], and mitigated NAFLD and its related factors in multiple animal experiments. [119]. A reduction in inflammation, modification of the gut microbiota, and increased expression of spermidine were observed with supplementation with *L. reuteri* ZJ617 [99] and *L. rhamnosus* FJSYC4‐1 and *L. reuteri* FGSZY33L6 in combination, which enhanced SCFA production reduced glucose and insulin levels, ameliorating metabolic syndrome [101]. *L. reuteri* V3401 supplementation in patients has been shown to improve disease condition [111]. *L. rhamnosus* hsryfm 1301 and GG strains have been shown to have anti‐NAFLD effects by reducing oxidative stress and inflammation in animal models [57, 58, 82, 83]. Similar results were obtained with *Lactobacillus coryniformis* supsp. *torquens* T3 supplementation and *L. reuteri* FYNLJ109L1 intervention [95, 97]. *Lactobacillus johnsonii* JNU3402, whose activity is mediated by the PKA‐SREBP1c pathway, while *L. fermentum* CQPC06 are other probiotic strains that are effective at treating NAFLD through modification of the gut microbiota [91, 98]. A combination of several *Lactobacillus* strains, including *L. acidophilus* BCMC 12130, *L. casei* subsp. BCMC 12313, and *L. lactis* BCMC 12451, in NAFLD patients attenuated disease conditions by decreasing the abundance of pathogenic gut bacteria and proinflammatory cytokines [103]. Improved liver damage and peroxidation were observed when mice were supplemented with *L. paracasei* CNCMI‐4034, *L. rhamnosus* CNCM I‐4036, and *Bifidobacterium breve* CNCM I‐4035 [104]. *L. fermentum* CRL1446, *L. rhamnosus* GG, and *Lactobacillus helveticus* HY7804 in combination with other microbes have also shown promising results in improving metabolic disorders [49, 92]. Taken together, these studies indicate that microbial metabolites assist in improving inflammation, liver damage, insulin resistance, and several other conditions linked to metabolic syndrome. To increase the production of these metabolites or SCFAs, the administration of *Lactobacillus* as a probiotic might be a promising approach. Considering these outcomes from preclinical studies (Table 1) and clinical trials (Table 2), *Lactobacillus* strains may be effective at treating metabolic syndrome and NAFLD.

## 5. Muscle Atrophy

Muscle atrophy is the loss of muscle mass, function, and strength [5]. It can occur because of various factors, such as age, obesity, and diabetes. The occurrence of muscle atrophy increases with age and is termed sarcopenia [3]. Mechanisms that promote sarcopenia include inflammation, immune senescence, anabolic resistance, decreased physical activity, and increased oxidative stress. In addition to diet modulation and exercise, probiotics are being used, as several studies have shown the regulation of the gut microbiota, production of SCFAs, anti‐inflammatory and anti‐reactive oxygen species (ROS) activity by their supplementation [28, 54] (Tables 1 and 2). Apart from probiotics, a naturally occurring flavonoid‐apigenin, which is an antioxidant primarily found in vegetables, such as parsley and celery, herbs, such as chamomile, and various fruits, such as oranges, plays a significant role in attenuating muscle atrophy [118, 120, 121]). Studies have demonstrated that apigenin is useful in obesity‐induced, inflammation‐induced, and age‐related muscle loss and degradation conditions [120, 121]. There are common pathways between apigenin and probiotics that lead to improvements in disease conditions, such as mitochondrial biogenesis, the Fox3a/atrogin1 pathway, the NF‐*κ*B pathway, and a reduction in oxidative stress by altering or increasing the balance in the gut microbiome, and the link is SCFAs produced by these beneficial microbes [118, 120, 121].

**Figure 4:**
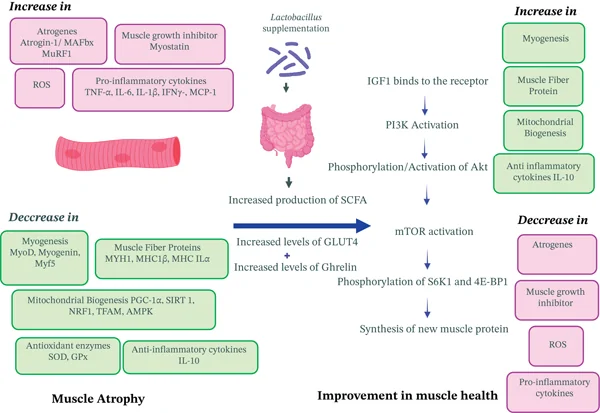
Proposed mechanisms by which *Lactobacillus* supplementation attenuates muscle atrophy and improves skeletal muscle health. *Lactobacillus* increases short‐chain fatty acid (SCFA) production and GLUT4 and ghrelin levels, activating the IGF‐1/PI3K/Akt/mTOR signaling pathway to increase muscle protein synthesis.

*L. paracasei* TD3 along with *Bacillus coagulans* T4 supplementation in mouse models has been useful for attenuating skeletal muscle atrophy and increasing muscle mass and strength. Major processes include the activation of the Akt pathway and a decrease in inflammatory markers [29, 51, 54]. Most animal studies also revealed a reduction in the expression of MuRF1, MAFbx/atrogin‐1, p16, TNF‐*α*, and IL‐6 [5]. Food fermented with *Lactobacillus curvatus* CP2998 targets GR (glucocorticoid receptor)‐dependent transcription, which is necessary for the upregulation of genes encoding E3 ubiquitin ligases, specifically MuRF1 and MAFbx (Atrogin‐1). These ligases drive the UPS, which breaks down myofibrillar proteins, resulting in muscle atrophy. Hence, direct suppression of GR‐dependent transcriptional activity prevents downstream processes and maintains muscle integrity [1]. Antlers fermented with strains of *L. curvatus* HY7602 have been shown to have similar effects on sarcopenic and muscle‐atrophied mice and myoblast cell lines by enhancing the synthesis of acetate and butyrate (SCFAs), which are believed to activate AMPK and PGC‐1*α*, which help regulate lipid, protein, and carbohydrate metabolism within skeletal muscle, enhancing muscle fiber regeneration and protein synthesis and suppressing muscle protein degradation [6, 13]. It has been shown that *Lactobacillus gasseri* CBT LGA2 ameliorates muscle atrophy through the same pathway [55]. Strains of *L. paracasei* and *L. casei L. casei* LC122, along with *Bifidobacterium longum* BL986, increase the abundance of *B. longum*, which naturally decreases with age and decreases the expression of proinflammatory cytokines and the expression of antioxidant enzymes in muscle tissue [24]. *L. paracasei* PS23 supplementation increases the expression of anti‐inflammatory cytokines and reduces the expression of proinflammatory cytokines, which helps reduce muscle wasting. Another major observation was the reduction in ROS levels and the regulation of genes involved in mitochondrial biogenesis, such as PGC1*α*, SIRT1, NRF1, and TFAM (mitochondrial transcription factor). These genes are downregulated in cases of atrophy, and probiotic supplementation enhances their expression, contributing to improved disease conditions [51]. In another animal model, along with mitochondrial biogenesis, the role of ghrelin, a gut hormone that influences muscle health, was observed. Probiotic intervention increased the expression of ghrelin in gut and muscle cells, likely by enhancing the phosphorylation of Akt, a critical component of the IGF1‐Akt pathway, which promotes cell survival and growth in skeletal muscle, leading to improved muscle health [12]. The combination of this strain with leucine and omega‐3 fatty acids reduced inflammation and increased plasma levels of BCAAs, along with enhancing mitochondrial function [50]. The administration of *L. paracasei* P62 and *Bifidobacterium bifidum* P61 promoted the growth of beneficial gut bacteria and reduced the abundance of proinflammatory bacteria, leading to an increase in the activation of AKT and mTOR in the muscle, which are vital for myogenesis. Thus, myogenic factors such as MyHC (myosin heavy chain) and myogenin maintain muscle mass and strength. A reduction in MuRF1 and MAFbx is responsible for muscle protein degradation through the suppression of the expression of FOXO3a and NF‐*κ*B, which are essential factors in the induction of atrogenes, ultimately leading to the alleviation of muscle atrophy [5]. An in vitro study revealed that whey protein‐fermented *L. gasseri* IM13 has a preventative effect against muscle atrophy through the IGF‐1/PI3K/AKT signaling pathway. It activated the phosphorylation of PI3K, AKT, and mTOR, ultimately leading to muscle protein synthesis. It also inactivates MAFbx and MuRF1, which are responsible for muscle protein degradation through the phosphorylation of FOXO3a [55]. Similar changes have been reported across both animal models and human trials when *L. plantarum* HY7715 and TWK10 strains were supplemented, and an increase in the expression of MyoD was observed [3, 28, 52]. In addition to these changes, the role of hsp60, a mitochondrial protein and NF‐*κ*B activation were observed in the case of *L. fermentum* LF31 supplementation [56]. Additionally, *L. paragasseri* ameliorated muscle atrophy and reduced myostatin expression. Protein synthesis and Akt phosphorylation are increased [79]. *L. fermentum* MCC2759 and MCC2760 reduce proinflammatory cytokines and increase GLUT4 expression, associated with a decrease in pathogenic bacteria [68]. Sarcopenia is attenuated by a reduction in ROS and inflammation due to increased butyric acid levels when mice are supplemented with *L. casei* Shirota [54]. A recent study has shown positive results with *L. plantarum* MIRE_TS55 supplementation through the Akt/mTOR/FoxO signaling pathway, decreasing muscle protein degradation and increasing protein synthesis [57]. Another study with the *L. plantarum* beLP1 strain showed similar results in in vitro and in vivo models [53]. Although very limited, clinical trials (Table 2) have also supported the results of preclinical studies (Table 1) indicating that *Lactobacillus* strains effectively help ameliorate sarcopenia or muscle atrophy and increase muscle mass and strength and yield better results when combined with other beneficial bacteria. Most importantly, the connection between the gut microbiota and muscle cells is established by the production of SCFAs, which regulate the expression of genes involved in muscle protein synthesis and degradation. The common pathway includes the phosphorylation of Akt, mTOR activation, increased synthesis of muscle proteins, and decreased levels of atrogenes and muscle growth inhibitors, leading to muscle growth and a reduction in muscle loss or atrophy. A few studies, as mentioned above, suggest that these pathways are directly regulated without suggesting the role of SCFAs, but the exact mechanism has yet to be studied (Figures 3 and 4). These studies suggest that specific strains of *Lactobacillus* might help in maintaining skeletal muscle health and in the reduction of the severity of muscle diseases to a certain extent.

## 6. Conclusion

Skeletal muscle health is fundamental to overall human physiology and depends on adequate nutrition and regular physical activity. The growing interest in probiotics has revealed their potential in regulating muscle health, spurring investigations into specific bacterial strains for therapeutic applications. Accumulating evidence suggests that *Lactobacillus*, one of the most extensively studied probiotic genera, significantly benefits the management of muscle‐related disorders and enhances exercise performance. These conclusions are drawn predominantly from preclinical, animal‐based, and cell‐based models; corroborating evidence from human clinical trials remains limited to a small number of strains, and therapeutic implications for human muscle health should therefore be considered preliminary until confirmed in larger, well‐controlled clinical studies. *Lactobacillus* supplementation restored gut microbiota homeostasis by enriching beneficial bacteria while suppressing pathogenic species. The mechanisms underlying these effects include reducing inflammation and oxidative stress, promoting mitochondrial biogenesis, stimulating protein synthesis, and attenuating protein degradation in skeletal muscle. SCFAs are the link between gut microbiota modulation and improvements in skeletal muscle health, exercise performance, and related metabolic disorders. The majority of the studies suggest that these microbial metabolites regulate the Akt/mTOR/FoxO and AMPK‐PPAR*α* signaling pathways. Certain *Lactobacillus* strains found in fermented foods show particular promise as probiotics for preventing and treating muscle atrophy and associated metabolic disorders, as well as improving exercise capacity.

However, several important considerations temper these findings. Some studies have reported elevated levels of *Lactobacillus* under pathological conditions, highlighting the complexity of host–microbe interactions and the need for further investigation. Critical limitations include the strain‐specific nature of probiotic benefits, whereby not all bacteria within a single species confer equivalent advantages, and the paucity of human studies examining the efficacy and mechanisms of these probiotics. Furthermore, individual responses to probiotic supplementation vary considerably on the basis of dietary pattern, geographical location, genetic factors, and baseline gut microbiome composition. The influence of geographical location is particularly noteworthy, as it limits the generalizability of findings across diverse populations.

Future research must prioritize well‐designed human clinical trials that account for key variables, including geographical location, dietary habits, comorbid conditions, and baseline microbiome profiles. Such studies are essential for resolving current discrepancies, elucidating the precise mechanisms by which *Lactobacillus* influences muscle health, and optimizing probiotic interventions for clinical application. Only through this comprehensive approach can the full therapeutic potential of *Lactobacillus* for muscle health be realized.

## 7. Future Perspective

Despite the growing recognition that microbial metabolites mediate the gut–muscle axis, critical knowledge gaps remain. The molecular mechanisms through which specific metabolites, particularly SCFAs, modulate muscle protein synthesis and degradation pathways require systematic elucidation. Furthermore, the substantial interindividual variability in the gut microbial composition, driven by diet, genetics, geographical location, and age, complicates efforts to establish universal probiotic recommendations. A major limitation of the current research is the scarcity of well‐controlled human trials examining the efficacy of probiotic supplementation on muscle health outcomes.

To advance this field, future investigations should adopt several key priorities. First, research must examine how *Lactobacillus* supplementation affects muscle health across diverse populations, accounting for age‐related differences, dietary patterns, and geographical variations that influence baseline microbiome composition. Second, longitudinal studies with extended follow‐up periods are essential to determine whether the beneficial effects of specific lactobacilli strains persist over time and translate into meaningful improvements in overall health and functional capacity. Third, mechanistic studies employing multiomics approaches are needed to identify the specific metabolites and signaling pathways responsible for gut–muscle crosstalk. Finally, personalized approaches that consider individual microbiome profiles, genetic predispositions, and lifestyle factors may optimize therapeutic outcomes and enable tailored probiotic interventions for maintaining skeletal muscle health throughout the lifespan.

**Nomenclature**
ACOX: acyl‐coenzyme A oxidase
CPT1: carnitine palmitoyltransferase 1
EIMD: exercise‐induced muscle damage
F/B: Firmicutes/Bacteroides ratio
GLUT4: glucose transporter type 4
IL: interleukin
LAB: lactic acid bacteria
NAFLD: nonalcoholic fatty liver disease
SCFA: short‐chain fatty acid
TNF: tumor necrosis factor
UPS: ubiquitin proteasome system

## Author Contributions

**Bencia Janet** wrote the first draft and performed all the analyses for the generation of tables and figures. **Rutuparna Jena** wrote the draft and generated the tables. **B.S. Satish Rao** and **Budheswar Dehury** provided the resources and supervised and edited the review.

## Funding

The authors acknowledge the Anusandhan National Research Foundation (Sanction Order: ANRF/ECRG/2024/003501/LS) for providing the Prime Minister Early Career Research Grant to BD.

## Disclosure

All the authors read and approved the MS before submission.

## Ethics Statement

No animals or human subjects were used in the above research.

## Conflicts of Interest

The authors declare no conflicts of interest.

## Supporting information


**Supporting Information** Additional supporting information can be found online in the Supporting Information section. Figure S1: Skeletal muscle health is related to gut dysbiosis, and supplementation with different strains of *Lactobacillus* ameliorate disease conditions and improve exercise performance.

## Data Availability

Data sharing is not applicable to this article, as no datasets were generated or analyzed during the current study.

## References

[1] Katsuki R. , Sakata S. , Nakao R. , Oishi K. , and Nakamura Y. , *Lactobacillus curvatus* CP2998 Prevents Dexamethasone-Induced Muscle Atrophy in C2C12 Myotubes, Journal of Nutritional Science and Vitaminology. (2019) 65, no. 5, 455–458, 10.3177/jnsv.65.455, 31666484. 31666484

[2] Zhong S. , Sun Z. , Tian Q. , Wen W. , Chen F. , Huang X. , and Li Y. , *Lactobacillus delbrueckii* Alleviates Lipopolysaccharide-Induced Muscle Inflammation and Atrophy in Weaned Piglets Associated With Inhibition of Endoplasmic Reticulum Stress and Protein Degradation, FASEB Journal. (2024) 38, no. 17, e70041, 10.1096/fj.202400969RR, 39250170. 39250170

[3] Lee K. , Kim J. , Park S.-D. , Shim J.-J. , and Lee J.-L. , *Lactobacillus plantarum* HY7715 Ameliorates Sarcopenia by Improving Skeletal Muscle Mass and Function in Aged Balb/c Mice, International Journal of Molecular Sciences. (2021) 22, no. 18, 10023, 10.3390/ijms221810023, 34576187. PMC8466743 34576187

[4] Yeh W.-L. , Hsu Y.-J. , Ho C.-S. , Ho H.-H. , Kuo Y.-W. , Tsai S.-Y. , Huang C.-C. , and Lee M.-C. , *Lactobacillus plantarum* PL-02 Supplementation Combined With Resistance Training Improved Muscle Mass, Force, and Exercise Performance in Mice, Frontiers in Nutrition. (2022) 9, 10.3389/fnut.2022.896503, 35571912. PMC9094439 35571912

[5] Baek J.-S. , Shin Y.-J. , Ma X. , Park H.-S. , Hwang Y.-H. , and Kim D.-H. , *Bifidobacterium bifidum* and *Lactobacillus paracasei* Alleviate Sarcopenia and Cognitive Impairment in Aged Mice by Regulating Gut Microbiota-Mediated AKT, NF-*κ*B, and FOXO3a Signaling Pathways, Immunity & Ageing. (2023) 20, no. 1, 10.1186/s12979-023-00381-5, 37872562. PMC10591382 37872562

[6] Jeon H. , Lee K. , Kim J.-Y. , Shim J.-J. , and Lee J.-L. , Effect of *Lactobacillus curvatus* HY7602-Fermented Antler on Sarcopenia in Mice, Fermentation. (2023) 9, no. 5, 10.3390/fermentation9050429.

[7] Hou Y. , Xiang J. , Wang B. , Duan S. , Song R. , Zhou W. , Tan S. , and He B. , Pathogenesis and Comprehensive Treatment Strategies of Sarcopenia in Elderly Patients With Type 2 Diabetes Mellitus, Frontiers in Endocrinology. (2024) 14, 1263650, 10.3389/fendo.2023.1263650, 38260146. PMC10801049 38260146

[8] Patel M. G. , Makwana H. H. , and Kalariya H. , Unraveling the Enigma of Sarcopenia and Sarcopenic Obesity in Indian Adults With Type 2 Diabetes – A Comparative Cross-Sectional Study, Clinical Diabetes and Endocrinology. (2024) 10, no. 1, 10.1186/s40842-024-00179-4, 38880930. PMC11181647 38880930

[9] Pechmann L. M. , Jonasson T. H. , Canossa V. S. , Trierweiler H. , Kisielewicz G. , Petterle R. R. , Moreira C. A. , and Borba V. Z. C. , Sarcopenia in Type 2 Diabetes Mellitus: A Cross-Sectional Observational Study, International Journal of Endocrinology. (2020) 2020, 9, 10.1155/2020/7841390, 33178269. PMC7644336 33178269

[10] Wang L. , He X. , Zhang Z. , and Chen N. , Distinct Gut Microbiota Signatures in Order People With Sarcopenia Without Obesity and Sarcopenic Obesity, 2024, preprint, Research Square., May 30, 2024 10.21203/rs.3.rs-4407157/v1.

[11] Wang L. , Meng F.-J. , Jin Y.-H. , Wu L.-Q. , Tang R.-Y. , Xu K.-H. , Guo Y. , Mao J.-J. , Ding J.-P. , and Li J. , Effects of Probiotic Supplementation on 12 min Run Performance, Mood Management, Body Composition and Gut Microbiota in Amateur Marathon Runners: A Double-Blind Controlled Trial, Journal of Exercise Science & Fitness. (2024) 22, no. 4, 297–304, 10.1016/j.jesf.2024.04.004, 38706951. PMC11066675 38706951

[12] Cheng L.-H. , Cheng S.-H. , Wu C.-C. , Huang C.-L. , Wen P.-J. , Chang M.-Y. , and Tsai Y.-C. , *Lactobacillus paracasei* PS23 Dietary Supplementation Alleviates Muscle Aging via Ghrelin Stimulation in d-Galactose-Induced Aging Mice, Journal of Functional Foods. (2021) 85, 104651, 10.1016/j.jff.2021.104651.

[13] Jeon H. , Kim Y.-T. , Jang W. Y. , Kim J.-Y. , Heo K. , Shim J.-J. , Lee J.-L. , Yang D.-C. , and Kang S. C. , Effects of *Lactobacillus curvatus* HY7602-Fermented Antlers in Dexamethasone-Induced Muscle Atrophy, Fermentation. (2022) 8, no. 9, 10.3390/fermentation8090454.

[14] Lee J. , Kang M. , Yoo J. , Lee S. , Kang M. , Yun B. , Kim J. N. , Moon H. , Chung Y. , and Oh S. , *Lactobacillus rhamnosus* JY02 Ameliorates Sarcopenia by Anti-Atrophic Effects in a Dexamethasone-Induced Cellular and Murine Model, Journal of Microbiology and Biotechnology. (2023) 33, no. 7, 915–925, 10.4014/jmb.2303.03001, 36998149. PMC10394339 36998149

[15] Lee M.-C. , Hsu Y.-J. , Chen M.-T. , Kuo Y.-W. , Lin J.-H. , Hsu Y.-C. , Huang Y.-Y. , Li C.-M. , Tsai S.-Y. , Hsia K.-C. , Ho H.-H. , and Huang C.-C. , Efficacy of *Lactococcus lactis* subsp. *lactis* LY-66 and *Lactobacillus plantarum* PL-02 in Enhancing Explosive Strength and Endurance: A Randomized, Double-Blinded Clinical Trial, Nutrients. (2024) 16, no. 12, 10.3390/nu16121921, 38931275. PMC11206817 38931275

[16] Lee M.-C. , Ho C.-S. , Hsu Y.-J. , and Huang C.-C. , Live and Heat-Killed Probiotic *Lactobacillus paracasei* PS23 Accelerated the Improvement and Recovery of Strength and Damage Biomarkers After Exercise-Induced Muscle Damage, Nutrients. (2022) 14, no. 21, 10.3390/nu14214563, 36364825. PMC9658587 36364825

[17] Lee M.-C. , Hsu Y.-J. , Ho H. , Kuo Y. , Lin W.-Y. , Tsai S.-Y. , Chen W.-L. , Lin C.-L. , and Huang C.-C. , Effectiveness of Human-Origin *Lactobacillus plantarum* PL-02 in Improving Muscle mass, exercise performance and anti-fatigue, Exercise Performance and Anti-Fatigue. Scientific Reports. (2021) 11, no. 1, 19469, 10.1038/s41598-021-98958-x, 34593921. PMC8484333 34593921

[18] He P. , Du G. , Qin X. , and Li Z. , Reduced Energy Metabolism Contributing to Aging of Skeletal Muscle by Serum Metabolomics and Gut Microbiota Analysis, Life Sciences. (2023) 323, 121619, 10.1016/j.lfs.2023.121619, 36965523. 36965523

[19] Liu D. , Liu D. C. , Fan H. , and Wang Y. , *Lactobacillus fermentum* CQPC08 Attenuates Exercise-Induced Fatigue in Mice Through Its Antioxidant Effects and Effective Intervention of Galactooligosaccharide, Drug Design, Development and Therapy. (2021) 15, 5151–5164, 10.2147/DDDT.S317456, 34992351. PMC8714972 34992351

[20] Mahdieh M. S. , Maryam J. , Bita B. , Neda F. , Motahare M. , Mahboobeh B. , Quinn LeBris S. , and Kalani Behrooz S. , A Pilot Study On The Relationship Between Lactobacillus, Bifidibactrium Counts And Inflammatory Factors Following Exercise Training, Archives of Physiology and Biochemistry. (2023) 129, no. 3, 778–787, 10.1080/13813455.2021.1871763, 33455471. 33455471

[21] Dodero V. I. , Morre S. A. , and Behzadi P. , Editorial: Gut Microbiota And Immunity In Health And Disease: Dysbiosis And Eubiosis’s Effects On The Human Body, Frontiers in Immunology. (2024) 15, 1536258, 10.3389/fimmu.2024.1536258, 39742257. PMC11685867 39742257

[22] Gizard F. , Fernandez A. , and De Vadder F. , Interactions Between Gut Microbiota and Skeletal Muscle, Nutrition and Metabolic Insights. (2020) 13, 1178638820980490, 10.1177/1178638820980490, 33402830. PMC7745561 33402830

[23] Ho P. Y. , Chou Y. C. , Koh Y. C. , Lin W. S. , Chen W. J. , Tseng A. L. , Gung C. L. , Wei Y. S. , and Pan M. H. , *Lactobacillus* rhamnosus 069 and *Lactobacillus brevis* 031: Unraveling Strain-Specific Pathways for Modulating Lipid Metabolism and Attenuating High-Fat-Diet-Induced Obesity in Mice, ACS Omega. (2024) 9, no. 26, 28520–28533, 10.1021/acsomega.4c02514, 38973907. PMC11223209 38973907

[24] Ni Y. , Yang X. , Zheng L. , Wang Z. , Wu L. , Jiang J. , Yang T. , Ma L. , and Fu Z. , *Lactobacillus* and *Bifidobacterium* Improves Physiological Function and Cognitive Ability in Aged Mice by the Regulation of Gut Microbiota, Molecular Nutrition & Food Research. (2019) 63, no. 22, e1900603, 10.1002/mnfr.201900603, 31433910. 31433910

[25] Clementino J. R. , de Oliveira L. I. G. , Salgaço M. K. , de Oliveira F. L. , Mesa V. , Tavares J. F. , Silva-Pereira L. , Raimundo B. V. B. , Oliveira K. C. , Medeiros A. I. , Silva F. A. , Sivieri K. , and Magnani M. , *β*-Glucan Alone or Combined with *Lactobacillus acidophilus* Positively Influences the Bacterial Diversity and Metabolites in the Colonic Microbiota of Type II Diabetic Patients, Probiotics and Antimicrobial Proteins. (2025) 17, no. 5, 3728–3742, 10.1007/s12602-025-10491-9. 40011383

[26] Yang Y. , Wang Y. , Cao X. , Shi L. , and Wang Y. , *Lactobacillus buchneri* Ameliorates Obesity-Related Disorders Induced by High-Fat and High-Cholesterol Diet in Mice, Food and Humanity. (2024) 3, 100317, 10.1016/j.foohum.2024.100317.

[27] Yang Y. , Zhao Y. , and Lei H. , Alleviating Effect of *Lactobacillus rhamnosus* SDSP202418 on Exercise-Induced Fatigue in Mice, Frontiers in Microbiology. (2024) 15, 1420872, 10.3389/fmicb.2024.1420872, 39391603. PMC11464290 39391603

[28] Lee Y.-S. , Lee D. , Park G.-S. , Ko S.-H. , Park J. , Lee Y.-K. , and Kang J. , *Lactobacillus plantarum* HAC01 Ameliorates Type 2 Diabetes in High-Fat Diet and Streptozotocin-Induced Diabetic Mice in Association With Modulating the Gut Microbiota, Food & Function. (2021) 12, no. 14, 6363–6373, 10.1039/D1FO00698C, 34105563. 34105563

[29] Shams M. , Esmaeili F. , Sadeghi S. , Shanaki-Bavarsad M. , Seyyed Ebrahimi S. S. , Hashemnia S. M. R. , Tajabadi-Ebrahimi M. , Emamgholipour S. , and Shanaki M. , Bacillus coagulans T4 and *Lactobacillus* paracasei TD3 Ameliorate Skeletal Muscle Oxidative Stress and Inflammation in High-Fat Diet-Fed C57BL/6J Mice, Iranian Journal of Pharmaceutical Research. (2023) 22, no. 1, e135249, 10.5812/ijpr-135249, 38116571. PMC10728858 38116571

[30] Petakh P. , Duve K. , Oksenych V. , Behzadi P. , and Kamyshnyi O. , Molecular Mechanisms and Therapeutic Possibilities of Short-Chain Fatty Acids in Posttraumatic Stress Disorder Patients: A Mini-Review, Frontiers in Neuroscience. (2024) 18, 1394953, 10.3389/fnins.2024.1394953, 38887367. PMC11182003 38887367

[31] Mao X. , Lv K. , Qi W. , Cheng W. , Li T. , Sun Y. , Jin H. , Pan H. , and Wang D. , Research Progress on Sarcopenia in the Musculoskeletal System, Bone Research. (2025) 13, no. 1, 10.1038/s41413-025-00455-8, 40987776. PMC12457612 40987776

[32] Behzadi P. , Chandran D. , Chakraborty C. , Bhattacharya M. , Saikumar G. , Dhama K. , Chakraborty A. , Mukherjee S. , and Sarshar M. , The Dual Role of Toll-Like Receptors in COVID-19: Balancing Protective Immunity and Immunopathogenesis, International Journal of Biological Macromolecules. (2024) 284, 137836, 10.1016/j.ijbiomac.2024.137836. 39613064

[33] Behzadi P. , Sameer A. S. , Nissar S. , Banday M. Z. , Gajdács M. , García-Perdomo H. A. , Akhtar K. , Pinheiro M. , Magnusson P. , Sarshar M. , and Ambrosi C. , The Interleukin-1 (IL-1) Superfamily Cytokines and Their Single Nucleotide Polymorphisms (SNPs), Journal of Immunology Research. (2022) 2022, no. 1, 2054431, 10.1155/2022/2054431. PMC8976653 35378905

[34] Kang L. , Li P. , Wang D. , Wang T. , Hao D. , and Qu X. , Alterations In Intestinal Microbiota Diversity, Composition, And Function In Patients With Sarcopenia, Scientific Reports. (2021) 11, no. 1, 10.1038/s41598-021-84031-0, 33633246. PMC7907362 33633246

[35] Yu C.-H. , Lai C.-C. , Chen J.-H. , Chen I.-C. , Tai H.-L. , and Fu S.-K. , Effect of *Lactobacillus plantarum* PS128 on Neuromuscular Efficiency After a Half-Marathon, Frontiers in Physiology. (2023) 14, 1254985, 10.3389/fphys.2023.1254985, 38098805. PMC10720321 38098805

[36] Zhu J. , Peng F. , Yang H. , Luo J. , Zhang L. , Chen X. , Liao H. , Lei H. , Liu S. , Yang T. , and Luo G. , Probiotics And Muscle Health: The Impact Of Lactobacillus On Sarcopenia Through The Gut-Muscle Axis, Frontiers in Microbiology. (2025) 16, 1559119, 10.3389/fmicb.2025.1559119, 40160272. PMC11952772 40160272

[37] Herrera-Rocha K. M. , Manjarrez-Juanes M. M. , Larrosa M. , Barrios-Payán J. A. , Rocha-Guzmán N. E. , Macías-Salas A. , Gallegos-Infante J. A. , Álvarez S. A. , González-Laredo R. F. , and Moreno-Jiménez M. R. , The Synergistic Effect of Quince Fruit and Probiotics (*Lactobacillus* and *Bifidobacterium*) on Reducing Oxidative Stress and Inflammation at the Intestinal Level and Improving Athletic Performance During Endurance Exercise, Nutrients. (2023) 15, no. 22, 10.3390/nu15224764, 38004161. PMC10675360 38004161

[38] Huang W.-C. , Lee M.-C. , Lee C.-C. , Ng K.-S. , Hsu Y.-J. , Tsai T.-Y. , Young S.-L. , Lin J.-S. , and Huang C.-C. , Effect of *Lactobacillus plantarum* TWK10 on Exercise Physiological Adaptation, Performance, and Body Composition in Healthy Humans, Nutrients. (2019) 11, no. 11, 10.3390/nu11112836, 31752370. PMC6893516 31752370

[39] Fu S.-K. , Tseng W.-C. , Tseng K.-W. , Lai C.-C. , Tsai Y.-C. , Tai H.-L. , and Hsu C.-C. , Effect of Daily Oral *Lactobacillus plantarum* PS128 on Exercise Capacity Recovery After a Half-Marathon, Nutrients. (2021) 13, no. 11, 10.3390/nu13114023, 34836278. PMC8619570 34836278

[40] Huang W.-C. , Wei C.-C. , Huang C.-C. , Chen W.-L. , and Huang H.-Y. , The Beneficial Effects of *Lactobacillus plantarum* PS128 on High-Intensity, Exercise-Induced Oxidative Stress, Inflammation, and Performance in Triathletes, Nutrients. (2019) 11, no. 2, 10.3390/nu11020353, 30736479. PMC6412901 30736479

[41] Maymandinejad I. , Hemmatinafar M. , Jäger R. , Imanian B. , Koushkie Jahromi M. , and Suzuki K. , Synergistic Effects of Probiotic and Omega-3 Supplementation With Ultra-Short Race Pace Training on Sprint Swimming Performance, Nutrients. (2025) 17, no. 14, 10.3390/nu17142296, 40732922. PMC12300206 40732922

[42] Chen Y.-M. , Wei L. , Chiu Y.-S. , Hsu Y.-J. , Tsai T.-Y. , Wang M.-F. , and Huang C.-C. , *Lactobacillus plantarum* TWK10 Supplementation Improves Exercise Performance and Increases Muscle Mass in Mice, Nutrients. (2016) 8, no. 4, 10.3390/nu8040205, 27070637. PMC4848674 27070637

[43] Chen Y. , Liao C. , Huang Y. , Chen M. , Huang C. , Chen W. , and Chiu Y. , Proteome and Microbiota Analysis Highlight *Lactobacillus* plantarum TWK10 Supplementation Improves Energy Metabolism and Exercise Performance in Mice, Food Science & Nutrition. (2020) 8, no. 7, 3525–3534, 10.1002/fsn3.1635. PMC7382123 32724615

[44] Chen Q. , Liu C. , Zhang Y. , Wang S. , and Li F. , Effect of *Lactobacillus plantarum* KSFY01 on the Exercise Capacity of D-Galactose-Induced Oxidative Stress-Aged Mice, Frontiers in Microbiology. (2022) 13, 1030833, 10.3389/fmicb.2022.1030833, 36620024. PMC9812958 36620024

[45] Yi R. , Feng M. , Chen Q. , Long X. , Park K.-Y. , and Zhao X. , The Effect of *Lactobacillus plantarum* CQPC02 on Fatigue and Biochemical Oxidation Levels in a Mouse Model of Physical Exhaustion, Frontiers in Nutrition. (2021) 8, 10.3389/fnut.2021.641544, 34095185. PMC8173150 34095185

[46] Lee M.-C. , Hsu Y.-J. , Ho H.-H. , Hsieh S.-H. , Kuo Y.-W. , Sung H.-C. , and Huang C.-C. , *Lactobacillus salivarius* Subspecies *salicinius* SA-03 Is a New Probiotic Capable of Enhancing Exercise Performance and Decreasing Fatigue, Microorganisms. (2020) 8, no. 4, 10.3390/microorganisms8040545, 32283729. PMC7232535 32283729

[47] Cai L. and Wang B. , Regulation of Colon Injury and Improvement of Exercise Performance in Exhausted Running Mice by *Lactobacillus pentosus* CQZC02, Frontiers in Physiology. (2024) 15, 1475413, 10.3389/fphys.2024.1475413, 39371599. PMC11450257 39371599

[48] Tsai Y. S. , Hsu Y. J. , Huang C. C. , Lin S. W. , Chen Y. L. , and Chen C. C. , Probiotic Lactobacillus Rhamnosus GKLC1 Enhances Endurance And Metabolism In Mice With Low Exercise Capacity, BMC Research Notes. (2026) 19, no. 1, 10.1186/s13104-026-07757-y, 41792776. PMC13077920 41792776

[49] Kim H. , Lee K. , Kim J.-Y. , Shim J.-J. , Lim J. , Kim J.-Y. , and Lee J.-L. , *Lactobacillus helveticus* Isolated From Raw Milk Improves Liver Function, Hepatic Steatosis, and Lipid Metabolism in Non-Alcoholic Fatty Liver Disease Mouse Model, Microorganisms. (2023) 11, no. 10, 10.3390/microorganisms11102466, 37894124. PMC10609090 37894124

[50] Rondanelli M. , Gasparri C. , Barrile G. C. , Battaglia S. , Cavioni A. , Giusti R. , Mansueto F. , Moroni A. , Nannipieri F. , Patelli Z. , Razza C. , Tartara A. , and Perna S. , Effectiveness of a Novel Food Composed of Leucine, Omega-3 Fatty Acids and Probiotic *Lactobacillus paracasei* PS23 for the Treatment of Sarcopenia in Elderly Subjects: A 2-Month Randomized Double-Blind Placebo-Controlled Trial, Nutrients. (2022) 14, no. 21, 10.3390/nu14214566, 36364828. PMC9656258 36364828

[51] Chen L. H. , Huang S. Y. , Huang K. C. , Hsu C. C. , Yang K. C. , Li L. A. , Chan C. H. , and Huang H. Y. , *Lactobacillus paracasei* PS23 Decelerated Age-Related Muscle Loss by Ensuring Mitochondrial Function in SAMP8 Mice, Aging. (2019) 11, no. 2, 756–770, 10.18632/AGING.101782, 30696799. PMC6366975 30696799

[52] Lee M.-C. , Tu Y.-T. , Lee C.-C. , Tsai S.-C. , Hsu H.-Y. , Tsai T.-Y. , Liu T.-H. , Young S.-L. , Lin J.-S. , and Huang C.-C. , *Lactobacillus plantarum* TWK10 Improves Muscle Mass and Functional Performance in Frail Older Adults: A Randomized, Double-Blind Clinical Trial, Microorganisms. (2021) 9, no. 7, 10.3390/microorganisms9071466, 34361902. PMC8305125 34361902

[53] Choi S.-I. , You S. , Kim S. , Won G. , Kang C.-H. , and Kim G.-H. , Weissella cibaria MG5285 and *Lactobacillus reuteri* MG5149 Attenuated Fat Accumulation in Adipose and Hepatic Steatosis in High-Fat Diet-Induced C57BL/6J Obese Mice, Food & Nutrition Research. (2021) 65, 10-29219, 10.29219/fnr.v65.8087, 34776827. PMC8559444 34776827

[54] Chen L. , Chang S. , Chang H. , Wu C. , Pan C. , Chang C. , Chan C. , and Huang H. , Probiotic Supplementation Attenuates Age-Related Sarcopenia via the Gut–Muscle Axis in SAMP8 Mice, Journal of Cachexia, Sarcopenia and Muscle. (2022) 13, no. 1, 515–531, 10.1002/jcsm.12849, 34766473. PMC8818665 34766473

[55] Jang J. H. , Joung J. Y. , Pack S. P. , and Oh N. S. , Preventive Effect of Fermented Whey Protein Mediated by *Lactobacillus gasseri* IM13 via the PI3K/AKT/FOXO Pathway in Muscle Atrophy, Journal of Dairy Science. (2024) 107, no. 5, 2606–2619, 10.3168/jds.2023-24027, 37977441. 37977441

[56] Sausa M. , Paladino L. , Scalia F. , Zummo F. P. , Vergilio G. , Rappa F. , Cappello F. , Gratie M. I. , Proia P. , di Felice V. , and Marino Gammazza A. , Lactobacillus Fermentum LF31 Supplementation Reversed Atrophy Fibers In A Model Of Myopathy Through The Modulation Of IL-6, TNF-*Α*, And Hsp60 Levels Enhancing Muscle Regeneration, Nutrients. (2025) 17, no. 9, 10.3390/nu17091550, 40362856. PMC12073311 40362856

[57] Kim B. , Park K.-Y. , Ji Y. , Park S. , Holzapfel W. , and Hyun C.-K. , Protective Effects of *Lactobacillus rhamnosus* GG Against Dyslipidemia in High-Fat Diet-Induced Obese Mice, Biochemical and Biophysical Research Communications. (2016) 473, no. 2, 530–536, 10.1016/j.bbrc.2016.03.107, 27018382. 27018382

[58] Arellano-García L. , Macarulla M. T. , Cuevas-Sierra A. , Martínez J. A. , Portillo M. P. , and Milton-Laskibar I. , *Lactobacillus rhamnosus* GG Administration Partially Prevents Diet-Induced Insulin Resistance in Rats: A Comparison With Its Heat-Inactivated Parabiotic, Food and Function. (2023) 14, no. 19, 8865–8875, 10.1039/d3fo01307c, 37698059. 37698059

[59] Park K. , Kim B. , and Hyun C. , *Lactobacillus rhamnosus* GG Improves Glucose Tolerance Through Alleviating ER stress and Suppressing Macrophage Activation in db/db Mice, Journal of Clinical Biochemistry and Nutrition. (2015) 56, no. 3, 240–246, 10.3164/jcbn.14-116, 26060355. PMC4454087 26060355

[60] Wu Z. , Gao J. , Yu C. , Zhao W. , Chen N. , Valencak T. G. , and Ren D. , *Lactobacillus rhamnosus* ZJUIDS07 Ameliorates Type 2 Diabetes in Mice Through the Microbiome-Gut-Pancreas Axis, Food Bioscience. (2024) 62, 105297, 10.1016/j.fbio.2024.105297.

[61] Gu Y. , Chen H. , Li X. , Li D. , Sun Y. , Yang L. , Ma Y. , and Chan E. C. Y. , *Lactobacillus paracasei* IMC502 Ameliorates Type 2 Diabetes by Mediating Gut microbiota–SCFA–hormone/inflammation Pathway in Mice, Journal of the Science of Food and Agriculture. (2023) 103, no. 6, 2949–2959, 10.1002/jsfa.12267, 36221226. 36221226

[62] Guilbaud A. , Howsam M. , Niquet-Léridon C. , Delguste F. , Fremont M. , Lestavel S. , Maboudou P. , Garat A. , Schraen S. , Onraed B. , Foligné B. , Boulanger É. , and Tessier F. J. , The Effect of *Lactobacillus fermentum* ME-3 Treatment on Glycation and Diabetes Complications, Molecular Nutrition & Food Research. (2020) 64, no. 6, e1901018, 10.1002/mnfr.201901018, 31991062. 31991062

[63] Pakaew K. , Chonpathompikunlert P. , Wongmanee N. , Rojanaverawong W. , Sitdhipol J. , Thaveethaptaikul P. , Charoenphon N. , and Hanchang W. , *Lactobacillus reuteri* TISTR 2736 Alleviates Type 2 Diabetes in Rats via the Hepatic IRS1/PI3K/AKT Signaling Pathway by Mitigating Oxidative Stress and Inflammatory Mediators, European Journal of Nutrition. (2025) 64, no. 1, 10.1007/s00394-024-03529-1, 39589518. 39589518

[64] Sharma P. , Bhardwaj P. , and Singh R. , Administration of *Lactobacillus casei* and *Bifidobacterium bifidum* Ameliorated Hyperglycemia, Dyslipidemia, and Oxidative Stress in Diabetic Rats, International Journal of Preventive Medicine. (2016) 7, no. 1, 10.4103/2008-7802.188870, 27625767. PMC5007903 27625767

[65] Yan F. , Li N. , Shi J. , Li H. , Yue Y. , Jiao W. , Wang N. , Song Y. , Huo G. , and Li B. , *Lactobacillus acidophilus* Alleviates Type 2 Diabetes by Regulating Hepatic Glucose, Lipid Metabolism and Gut Microbiota in Mice, Food & Function. (2019) 10, no. 9, 5804–5815, 10.1039/C9FO01062A, 31461095. 31461095

[66] Balakumar M. , Prabhu D. , Sathishkumar C. , Prabu P. , Rokana N. , Kumar R. , Raghavan S. , Soundarajan A. , Grover S. , Batish V. K. , Mohan V. , and Balasubramanyam M. , Improvement in Glucose Tolerance and Insulin Sensitivity by Probiotic Strains of Indian Gut Origin in High-Fat Diet-Fed C57BL/6J Mice, European Journal of Nutrition. (2018) 57, no. 1, 279–295, 10.1007/s00394-016-1317-7, 27757592. 27757592

[67] Farida E. , Nuraida L. , Giriwono P. E. , and Jenie B. S. L. , *Lactobacillus rhamnosus* Reduces Blood Glucose Level Through Downregulation of Gluconeogenesis Gene Expression in Streptozotocin-Induced Diabetic Rats, International Journal of Food Science. (2020) 2020, 12, 10.1155/2020/6108575, 32399477. PMC7201496 32399477

[68] Archer A. C. , Muthukumar S. P. , and Halami P. M. , *Lactobacillus fermentum* MCC2759 and MCC2760 Alleviate Inflammation and Intestinal Function in High-Fat Diet-Fed and Streptozotocin-Induced Diabetic Rats, Probiotics and Antimicrobial Proteins. (2021) 13, no. 4, 1068–1080, 10.1007/s12602-021-09744-0, 33575913. PMC8342349 33575913

[69] Ji Y. , Park S. , Park H. , Hwang E. , Shin H. , Pot B. , and Holzapfel W. H. , Modulation of Active Gut Microbiota by *Lactobacillus rhamnosus* GG in a Diet Induced Obesity Murine Model, Frontiers in Microbiology. (2018) 9, 10.3389/fmicb.2018.00710, 29692770. PMC5902571 29692770

[70] Li X. , Huang Y. , Song L. , Xiao Y. , Lu S. , Xu J. , Li J. , and Ren Z. , *Lactobacillus plantarum* Prevents Obesity via Modulation of Gut Microbiota and Metabolites in High-Fat Feeding Mice, Journal of Functional Foods. (2020) 73, 104103, 10.1016/j.jff.2020.104103.

[71] Pan R. , Wang T. , Bai J. , Zhang J. , Gu Y. , Zhao Z. , Tang R. , Qian Z. , Yan L. , Xiao X. , Liang S. , and Dong Y. , *Lactobacillus plantarum* fermented Barley Extract Attenuates Obesity in HFD‐induced Obese Rats by Regulating Gut Microbiota, Lipids. (2025) 60, no. 4, 237–248, 10.1002/lipd.12435, 40052274. 40052274

[72] Yoshitake R. , Hirose Y. , Murosaki S. , and Matsuzaki G. , Heat-Killed *Lactobacillus plantarum* L-137 Attenuates Obesity and Associated Metabolic Abnormalities in C57BL/6 J Mice on a High-Fat Diet, Bioscience of Microbiota, Food and Health. (2021) 40, no. 2, 84–91, 10.12938/bmfh.2020-040, 33996364. PMC8099634 33996364

[73] Huang E. , Kim S. , Park H. , Park S. , Ji Y. , Todorov S. D. , Lim S.-D. , and Holzapfel W. H. , Modulation of the Gut Microbiome and Obesity Biomarkers by *Lactobacillus plantarum* KC28 in a Diet-Induced Obesity Murine Model, Probiotics and Antimicrobial Proteins. (2021) 13, no. 3, 677–697, 10.1007/s12602-020-09720-0, 33188637. 33188637

[74] He Q. , Zhang Y. , Ma D. , Zhang W. , and Zhang H. , *Lactobacillus* casei Zhang Exerts Anti-Obesity Effect to Obese Glut 1 and Gut-Specific-Glut 1 Knockout Mice via Gut Microbiota Modulation Mediated Different Metagenomic Pathways, European Journal of Nutrition. (2022) 61, no. 4, 2003–2014, 10.1007/s00394-021-02764-0, 34984487. 34984487

[75] Park S.-S. , Lee Y.-J. , Song S. , Kim B. , Kang H. , Oh S. , and Kim E. , *Lactobacillus acidophilus* NS1 Attenuates Diet-Induced Obesity and Fatty Liver, Journal of Endocrinology. (2018) 237, no. 2, 87–100, 10.1530/JOE-17-0592, 29507043. 29507043

[76] Kim D. , Jeong D. , Kang I. , Kim H. , Song K. , and Seo K. , Dual Function of *Lactobacillus kefiri* DH5 in Preventing High-Fat-Diet-Induced Obesity: Direct Reduction of Cholesterol and Upregulation of PPAR-*α* in Adipose Tissue, Molecular Nutrition & Food Research. (2017) 61, no. 11, 10.1002/mnfr.201700252. 28691342

[77] Li P. , He X. , Feng E. , Wei J. , Tu H. , and Chen T. , *Lactobacillus acidophilus* JYLA-126 Ameliorates Obesity-Associated Metabolic Disorders by Positively Regulating the AMPK Signaling Pathway Through the Gut-Liver Axis, Probiotics and Antimicrobial Proteins. (2025) 17, no. 1, 62–80, 10.1007/s12602-023-10190-3, 38051435. 38051435

[78] Zhao C. , Xie L. , Shen J. , He H. , Zhang T. , Hao L. , Sun C. , Zhang X. , Chen M. , Liu F. , Li Z. , and Wang N. , *Lactobacillus acidophilus* YL01 and Its Exopolysaccharides Ameliorate Obesity and Insulin Resistance in Obese Mice via Modulating Intestinal Specific Bacterial Groups and AMPK/ACC Signaling Pathway, International Journal of Biological Macromolecules. (2025) 300, 140287, 10.1016/j.ijbiomac.2025.140287, 39863204. 39863204

[79] Kim M.-J. , Shin S.-K. , Han J.-W. , Kim J. E. , Lee M. J. , Bae H. R. , and Kwon E.-Y. , *Lactobacillus paragasseri* SBT2055 Attenuates Obesity via the Adipose Tissue-Muscle-Gut Axis in Obese Mice, Microbiological Research. (2025) 290, 127972, 10.1016/j.micres.2024.127972, 39566338. 39566338

[80] Jang H. , Joung H. , Chu J. , Cho M. , Kim Y.-W. , Kim K. H. , Shin C. H. , Lee J. , and Ha J.-H. , *Lactobacillus delbrueckii* Subsp. *Lactis* CKDB001 Ameliorates Metabolic Complications In High-Fat Diet-Induced Obese Mice, Nutrients. (2024) 16, no. 24, 10.3390/nu16244260. PMC11677567 39770882

[81] Hou S. , Li R. , Zhang Y. , Liang P. , Yang H. , He H. , Wang L. , Sun Y. , Jin T. , Liu Z. , and Xie J. , Supplementation of Mixed *Lactobacillus* Alleviates Metabolic Impairment, Inflammation, and Dysbiosis of the Gut Microbiota in an Obese Mouse Model, Frontiers in Nutrition. (2025) 12, 1554996, 10.3389/fnut.2025.1554996, 40206949. PMC11978641 40206949

[82] Yau Y. F. , El-Nezami H. , Galano J. , Kundi Z. M. , Durand T. , and Lee J. C. , *Lactobacillus rhamnosus* GG and Oat Beta-Glucan Regulated Fatty Acid Profiles Along the Gut-Liver-Brain Axis of Mice Fed With High Fat Diet and Demonstrated Antioxidant and Anti-Inflammatory Potentials, Molecular Nutrition & Food Research. (2020) 64, no. 18, e2000566, 10.1002/mnfr.202000566, 32780531. 32780531

[83] Chen D. , Liang Y. , Liang J. , Shen F. , Cheng Y. , Qu H. , Wa Y. , Guo C. , Gu R. , Qian J. , Chen X. , Zhang C. , and Guan C. , Beneficial Effects of *Lactobacillus rhamnosus hsryfm* 1301 Fermented Milk on Rats With Nonalcoholic Fatty Liver Disease, Journal of Dairy Science. (2023) 106, no. 3, 1533–1548, 10.3168/jds.2022-22383, 36710180. 36710180

[84] Wen X. , Liu H. , Luo X. , Lui L. , Fan J. , Xing Y. , Wang J. , Qiao X. , Li N. , and Wang G. , Supplementation of *Lactobacillus plantarum* ATCC14917 Mitigates Non-Alcoholic Fatty Liver Disease in High-Fat-Diet-Fed Rats, Frontiers in Microbiology. (2023) 14, 1146672, 10.3389/fmicb.2023.1146672, 37266005. PMC10229879 37266005

[85] Zhao L. , Shen Y. , Wang Y. , Wang L. , Zhang L. , Zhao Z. , and Li S. , *Lactobacillus plantarum* S9 Alleviates Lipid Profile, Insulin Resistance, and Inflammation in High-Fat Diet-Induced Metabolic Syndrome Rats, Scientific Reports. (2022) 12, no. 1, 15490, 10.1038/s41598-022-19839-5, 36109620. PMC9478128 36109620

[86] Kwon J. , Kim B. , Lee C. , Joung H. , Kim B.-K. , Choi I. S. , and Hyun C.-K. , Comprehensive Amelioration of High-Fat Diet-Induced Metabolic Dysfunctions Through Activation of the PGC-1*α* Pathway by Probiotics Treatment in Mice, PloS One. (2020) 15, no. 2, e0228932, 10.1371/journal.pone.0228932, 32040532. PMC7010303 32040532

[87] Cao F. , Ding Q. , Zhuge H. , Lai S. , Chang K. , Le C. , Yang G. , Valencak T. G. , Li S. , and Ren D. , *Lactobacillus plantarum* ZJUIDS14 Alleviates Non-Alcoholic Fatty Liver Disease in Mice in Association With Modulation in the Gut Microbiota, Frontiers in Nutrition. (2023) 9, 1071284, 10.3389/fnut.2022.1071284, 36698477. PMC9868733 36698477

[88] Wang Y. , Zhang Y. , Yang J. , Li H. , Wang J. , and Geng W. , *Lactobacillus plantarum* MA2 Ameliorates Methionine and Choline-Deficient Diet Induced Non-Alcoholic Fatty Liver Disease in Rats by Improving the Intestinal Microecology and Mucosal Barrier, Foods. (2021) 10, no. 12, 10.3390/foods10123126, 34945677. PMC8701163 34945677

[89] Zhao Z. , Chen L. , Zhao Y. , Wang C. , Duan C. , Yang G. , Niu C. , and Li S. , *Lactobacillus plantarum* NA136 Ameliorates Nonalcoholic Fatty Liver Disease by Modulating Gut Microbiota, Improving Intestinal Barrier Integrity, and Attenuating Inflammation, Applied Microbiology and Biotechnology. (2020) 104, no. 12, 5273–5282, 10.1007/s00253-020-10633-9, 32335723. 32335723

[90] Park E.-J. , Lee Y.-S. , Kim S. M. , Park G.-S. , Lee Y. H. , Jeong D. Y. , Kang J. , and Lee H.-J. , Beneficial Effects of *Lactobacillus plantarum* Strains on Non-Alcoholic Fatty Liver Disease in High Fat/High Fructose Diet-Fed Rats, Nutrients. (2020) 12, no. 2, 10.3390/nu12020542, 32093158. PMC7071439 32093158

[91] Mu J. , Tan F. , Zhou X. , and Zhao X. , *Lactobacillus fermentum* CQPC06 in Naturally Fermented Pickles Prevents Non-Alcoholic Fatty Liver Disease by Stabilizing the Gut–Liver Axis in Mice, Food & Function. (2020) 11, no. 10, 8707–8723, 10.1039/D0FO01823F, 32945305. 32945305

[92] Russo M. , Marquez A. , Herrera H. , Abeijon-Mukdsi C. , Saavedra L. , Hebert E. , Gauffin-Cano P. , and Medina R. , Oral Administration of *Lactobacillus fermentum* CRL1446 Improves Biomarkers of Metabolic Syndrome in Mice Fed a High-Fat Diet Supplemented With Wheat Bran, Food & Function. (2020) 11, no. 5, 3879–3894, 10.1039/D0FO00730G, 32421119. 32421119

[93] Hayashi H. , Sawada K. , Tanaka H. , Muro K. , Hasebe T. , Nakajima S. , Okumura T. , and Fujiya M. , The Effect of Heat-Killed *Lactobacillus brevis* SBL88 on Improving Selective Hepatic Insulin Resistance in Non-Alcoholic Fatty Liver Disease Mice Without Altering the Gut Microbiota, Journal of Gastroenterology and Hepatology. (2023) 38, no. 10, 1847–1854, 10.1111/jgh.16337, 37646384. 37646384

[94] Guo Z. , Yang S. , Qi L. , Ma X. , Wang Y. , Li B. , and He J. , *Lactobacillus acidophilus* KLDS1.0901 Ameliorates Non-Alcoholic Fatty Liver Disease by Modulating the Tryptophan Metabolite Indole-3-Aldehyde and Acting on Its Receptor AhR, Food & Function. (2025) 16, no. 12, 4939–4957, 10.1039/D4FO05280C, 40423690. 40423690

[95] Yang B. , Zheng F. , Stanton C. , Ross R. P. , Zhao J. , Zhang H. , and Chen W. , *Lactobacillus reuteri* FYNLJ109L1 Attenuating Metabolic Syndrome in Mice via Gut Microbiota Modulation and Alleviating Inflammation, Foods. (2021) 10, no. 9, 10.3390/foods10092081, 34574191. PMC8469823 34574191

[96] Werlinger P. , Nguyen H. T. , Gu M. , Cho J.-H. , Cheng J. , and Suh J.-W. , *Lactobacillus reuteri* MJM60668 Prevent Progression of Non-Alcoholic Fatty Liver Disease Through Anti-Adipogenesis and Anti-Inflammatory Pathway, Microorganisms. (2022) 10, no. 11, 10.3390/microorganisms10112203, 36363795. PMC9696116 36363795

[97] Song W. , Bai Y. Y. , Hu J. H. , Li L. L. , He W. W. , Liu C. C. , Li L. , Ning X. , Zhu L. N. , Cui X. L. , Chen B. , Wang T. Y. , Su K. X. , Miao Y. X. , Luo Y. E. , Sheng Q. L. , and Yue T. L. , *Lactobacillus coryniformis* subsp. *torquens* Inhibits Bone Loss in Obese Mice via Modification of the Gut Microbiota, Food & Function. (2023) 14, no. 10, 4522–4538, 10.1039/D2FO03863C, 37062959. 37062959

[98] Hong E. , Kang H. , Yang G. , Oh S. , and Kim E. , The PKA-SREBP1c Pathway Plays a Key Role in the Protective Effects of *Lactobacillus johnsonii* JNU3402 Against Diet-Induced Fatty Liver in Mice, Molecular Nutrition & Food Research. (2023) 67, no. 20, e2200496, 10.1002/mnfr.202200496, 37650271. 37650271

[99] Ma Y. , Zhong Y. , Tang W. , Valencak T. G. , Liu J. , Deng Z. , Mao J. , Liu D. , Wang S. , Wang Y. , and Wang H. , *Lactobacillus Reuteri* ZJ617 Attenuates Metabolic Syndrome via Microbiota-Derived Spermidine, Nature Communications. (2025) 16, no. 1, 10.1038/s41467-025-56105-4, 39837844. PMC11750987 39837844

[100] Wang J. , Tang H. , Zhang C. , Zhao Y. , Derrien M. , Rocher E. , Van-Hylckama Vlieg J. E. , Strissel K. , Zhao L. , Obin M. , and Shen J. , Modulation of Gut Microbiota During Probiotic-Mediated Attenuation of Metabolic Syndrome in High Fat Diet-Fed Mice, ISME Journal. (2015) 9, no. 1, 1–15, 10.1038/ismej.2014.99, 24936764. PMC4274436 24936764

[101] Zheng F. , Wang Z. , Stanton C. , Ross R. P. , Zhao J. , Zhang H. , Yang B. , and Chen W. , *Lactobacillus rhamnosus* FJSYC4-1 and *Lactobacillus reuteri* FGSZY33L6 Alleviate Metabolic Syndrome via Gut Microbiota Regulation, Food and Function. (2021) 12, no. 9, 3919–3930, 10.1039/d0fo02879g, 33977963. 33977963

[102] Khanna S. , Walia S. , Kondepudi K. K. , and Shukla G. , Administration of Indigenous Probiotics Modulate High-Fat Diet-Induced Metabolic Syndrome in Sprague Dawley Rats, Antonie van Leeuwenhoek. (2020) 113, no. 9, 1345–1359, 10.1007/s10482-020-01445-y, 32632629. 32632629

[103] Ayob N. , Muhammad Nawawi K. N. , Mohamad Nor M. H. , Raja Ali R. A. , Ahmad H. F. , Oon S. F. , and Mohd Mokhtar N. , The Effects of Probiotics on Small Intestinal Microbiota Composition, Inflammatory Cytokines and Intestinal Permeability in Patients With Non-Alcoholic Fatty Liver Disease, Biomedicines. (2023) 11, no. 2, 10.3390/biomedicines11020640, 36831176. PMC9953317 36831176

[104] Fontana L. , Plaza-Díaz J. , Robles-Bolívar P. , Valente-Godínez H. , Sáez-Lara M. J. , Abadía-Molina F. , Gómez-Llorente C. , Gil Á. , and Álvarez-Mercado A. I. , Bifidobacterium breve CNCM I-4035, *Lactobacillus paracasei* CNCM I-4034 and *Lactobacillus rhamnosus* CNCM I-4036 Modulate Macrophage Gene Expression and Ameliorate Damage Markers in the Liver of Zucker-Leprfa/fa Rats, Nutrients. (2021) 13, no. 1, 10.3390/nu13010202, 33440736. PMC7826559 33440736

[105] Yao F. , Jia R. , Huang H. , Yu Y. , Mei L. , Bai L. , Ding Y. , and Zheng P. , Effect of *Lactobacillus paracasei* N1115 and Fructooligosaccharides in Nonalcoholic Fatty Liver Disease, Archives of Medical Science. (2019) 15, no. 5, 1336–1344, 10.5114/aoms.2019.86611, 31572482. PMC6764303 31572482

[106] Huang W.-C. , Pan C.-H. , Wei C.-C. , and Huang H.-Y. , *Lactobacillus plantarum* PS128 Improves Physiological Adaptation and Performance in Triathletes through Gut Microbiota Modulation, Nutrients. (2020) 12, no. 8, 10.3390/nu12082315, 32752178. PMC7468698 32752178

[107] Bongiovanni T. , Santiago M. , Zielinska K. , Scheiman J. , Barsa C. , Jäger R. , Pinto D. , Rinaldi F. , Giuliani G. , Senatore T. , and Kostic A. D. , A *Lactobacillus consortium* Provides Insights Into the Sleep-Exercise-Microbiome Nexus in Proof of Concept Studies of Elite Athletes and in the General Population, Microbiome. (2025) 13, no. 1, 10.1186/s40168-024-01936-4, 39748236. PMC11697739 39748236

[108] Hsieh M.-C. , Tsai W.-H. , Jheng Y.-P. , Su S.-L. , Wang S.-Y. , Lin C.-C. , Chen Y.-H. , and Chang W.-W. , The Beneficial Effects of *Lactobacillus reuteri* ADR-1 or ADR-3 Consumption on Type 2 Diabetes Mellitus: A Randomized, Double-Blinded, Placebo-Controlled Trial, Scientific Reports. (2018) 8, no. 1, 16791, 10.1038/s41598-018-35014-1, 30429496. PMC6235926 30429496

[109] Lee S.-B. , Yoo B. , Baeg C. , Yun J. , Ryu D. , Kim G. , Kim S. , Shin H. , and Lee J. H. , A 12-Week, Randomized, Double-Blind, Placebo-Controlled Study to Evaluate the Efficacy and Safety of *Lactobacillus plantarum* LMT1-48 on Body Fat Loss, Nutrients. (2025) 17, no. 7, 10.3390/nu17071191, 40218949. PMC11990557 40218949

[110] Ji Y. , Chung Y. M. , Park S. , Jeong D. , Kim B. , and Holzapfel W. H. , Dose-Dependent and Strain-Dependent Anti-Obesity Effects of *Lactobacillus sakei* in a Diet Induced Obese Murine Model, PeerJ. (2019) 7, e6651, 10.7717/peerj.6651, 30923658. PMC6431538 30923658

[111] Tenorio-Jiménez C. , Martínez-Ramírez M. J. , Del Castillo-Codes I. , Arraiza-Irigoyen C. , Tercero-Lozano M. , Camacho J. , Chueca N. , García F. , Olza J. , Plaza-Díaz J. , Fontana L. , Olivares M. , Gil Á. , and Gómez-Llorente C. , *Lactobacillus reuteri* V3401 Reduces Inflammatory Biomarkers and Modifies the Gastrointestinal Microbiome in Adults With Metabolic Syndrome: The PROSIR Study, Nutrients. (2019) 11, no. 8, 10.3390/nu11081761, 31370223. PMC6723328 31370223

[112] Zhang X. , Chang Z. , Zhao S. , Wu X. , Wang X. , Ai G. , and Ning Z. , Effect Of Probiotic Intake On Athletic Ability In Healthy People: A Systematic Review And Bayesian Meta-Analysis, Frontiers in Nutrition. (2026) 13, 1731627, 10.3389/fnut.2026.1731627, 41693944. PMC12903275 41693944

[113] Ferenc K. , Jarmakiewicz-Czaja S. , and Filip R. , What Does Sarcopenia Have to Do With Nonalcoholic Fatty Liver Disease?, Life. (2024) 14, no. 1, 10.3390/life14010037, 38255652. PMC10820621 38255652

[114] Feng L. , Gao Q. , Hu K. , Wu M. , Wang Z. , Chen F. , Mei F. , Zhao L. , and Ma B. , Prevalence and Risk Factors of Sarcopenia in Patients With Diabetes: A Meta-Analysis, Journal of Clinical Endocrinology & Metabolism. (2022) 107, no. 5, 1470–1483, 10.1210/clinem/dgab884, 34904651. 34904651

[115] Tang C. , Tao J. , Sun J. , Lv F. , Lu Z. , and Lu Y. , Regulatory Mechanisms of Energy Metabolism and Inflammation in Oleic Acid-Treated HepG2 Cells from *Lactobacillus acidophilus* NX2‐6 Extract, Journal of Food Biochemistry. (2021) 45, no. 10, e13925, 10.1111/jfbc.13925, 34486133. 34486133

[116] Rezazadeh L. , Alipour B. , Jafarabadi M. A. , Behrooz M. , and Gargari B. P. , Daily Consumption Effects of Probiotic Yogurt Containing *Lactobacillus acidophilus* La5 and Bifidobacterium *lactis* Bb12 on Oxidative Stress in Metabolic Syndrome Patients, Clinical Nutrition ESPEN. (2021) 41, 136–142, 10.1016/j.clnesp.2020.12.003, 33487257. 33487257

[117] Zhu C. , Guan Q. , Song C. , Zhong L. , Ding X. , Zeng H. , Nie P. , and Song L. , Regulatory Effects Of Lactobacillus Fermented Black Barley On Intestinal Microbiota Of NAFLD Rats, Food Research International. (2021) 147, 110467, 10.1016/j.foodres.2021.110467. 34399465

[118] Wang W. , Li Q. , Chai W. , Sun C. , Zhang T. , Zhao C. , Yuan Y. , Wang X. , Liu H. , and Ye H. , *Lactobacillus paracasei* Jlus 66 Extenuate Oxidative Stress and Inflammation via Regulation of Intestinal Flora in Rats With Non Alcoholic Fatty Liver Disease, Food Science & Nutrition. (2019) 7, no. 8, 2636–2646, 10.1002/fsn3.1118, 31428351. PMC6694609 31428351

[119] Ye H. , Li Q. , Zhang Z. , Sun M. , Zhao C. , and Zhang T. , Effect of a Novel Potential Probiotic *Lactobacillus paracasei* Jlus 66 Isolated From Fermented Milk on Nonalcoholic Fatty Liver in Rats, Food & Function. (2017) 8, no. 12, 4539–4546, 10.1039/C7FO01108C, 29106426. 29106426

[120] de Castro J. M. , da Silva Melo A. , Silveira B. L. , Martins I. A. S. , Marçal M. M. , Dal Bosco T. , Keingeski M. B. , de Oliveira E. C. , Alvares-da-Silva M. R. , Türck P. , and da Rosa Araujo A. S. , Oral Apigenin Prevents Obesity-Related Muscular Atrophy, But Not Obesity Itself, In Middle-Aged Rats Fed A High-Calorie Diet, Biomedicine & Pharmacotherapy. (2025) 189, 118342, 10.1016/j.biopha.2025.118342, 40652723. 40652723

[121] Shiota C. , Abe T. , Kawai N. , Ohno A. , Teshima-Kondo S. , Mori H. , Terao J. , Tanaka E. , and Nikawa T. , Flavones Inhibit LPS-Induced Atrogin-1/Mafbx Expression In Mouse C2C12 Skeletal Myotubes, Journal Of Nutritional Science And Vitaminology. (2015) 61, no. 2, 188–194, 10.3177/jnsv.61.188, 26052151. 26052151

