# Supplementary material for: The Gut Microbiota–Muscle Axis: Lactobacillus as a Potential Modulator of Skeletal Muscle Health
Source: Biomed Res Int. 2026 Sep 22;2026:7133290. doi: 10.1155/bmri/7133290 (PMC13595175; doi:10.1155/bmri/7133290)
Supplement: Supplementary file 1 [file BMRI-2026-7133290-s001.docx]

**Supporting Information**

**The gut microbiota-muscle axis: *Lactobacillus* as a potential modulator of skeletal muscle health**

Bencia Janet^1,2^, Rutuparna Jena^2^, B. S. Satish Rao^1,*^, Budheswar Dehury^2,*^

^1^Department of Radiation Biology and Toxicology, Manipal School of Life Sciences, Manipal Academy of Higher Education, Manipal-576104, India

^2^Department of Bioinformatics, Manipal School of Life Sciences, Manipal Academy of Higher Education, Manipal-576104, India

***Corresponding Authors**

Dr. B. S. Satish Rao

Email: [rao.satish@manipal.edu](mailto:rao.satish@manipal.edu)

Dr. Budheswar Dehury

Email: [budheswar.dehury@manipal.edu](mailto:budheswar.dehury@manipal.edu), [budheswar.dehury@gmail.com](mailto:budheswar.dehury@gmail.com)

**Figure S1**. Skeletal muscle health is related to gut dysbiosis, and supplementation with different strains of *Lactobacillus* ameliorate disease conditions and improve exercise performance.
